# Meta-analysis of Genome Wide Association Studies Identifies Genetic Markers of Late Toxicity Following Radiotherapy for Prostate Cancer

**DOI:** 10.1016/j.ebiom.2016.07.022

**Published:** 2016-07-20

**Authors:** Sarah L. Kerns, Leila Dorling, Laura Fachal, Søren Bentzen, Paul D.P. Pharoah, Daniel R. Barnes, Antonio Gómez-Caamaño, Ana M. Carballo, David P. Dearnaley, Paula Peleteiro, Sarah L. Gulliford, Emma Hall, Kyriaki Michailidou, Ángel Carracedo, Michael Sia, Richard Stock, Nelson N. Stone, Matthew R. Sydes, Jonathan P. Tyrer, Shahana Ahmed, Matthew Parliament, Harry Ostrer, Barry S. Rosenstein, Ana Vega, Neil G. Burnet, Alison M. Dunning, Gillian C. Barnett, Catharine M.L. West, Radiogenomics Consortium

**Affiliations:** a Department of Radiation Oncology, University of Rochester Medical Center, Rochester, NY, USA; b Department of Radiation Oncology, Icahn School of Medicine at Mount Sinai, New York, NY, USA; c Department of Public Health and Primary Care, Centre for Cancer Genetic Epidemiology, Strangeways Research Laboratory, University of Cambridge, Cambridge CB1 8RN, UK; d Department of Oncology, Centre for Cancer Genetic Epidemiology, Strangeways Research Laboratory, University of Cambridge, Cambridge CB1 8RN, UK; e Grupo de Medicina Xenómica, Centro de Investigación Biomédica en Red de Enfermedades Raras (CIBERER), Universidade de Santiago de Compostela (USC), Santiago de Compostela, Spain; f Division of Biostatistics and Bioinformatics, University of Maryland Greenebaum Cancer Center, Baltimore, USA; g Department of Epidemiology and Public Health, University of Maryland School of Medicine, Baltimore, USA; h Department of Radiation Oncology, Complexo Hospitalario Universitario de Santiago, Servizo Galego de Saúde (SERGAS), Santiago de Compostela, Spain; i Joint Department of Physics, Institute of Cancer Research, Royal Marsden NHS Foundation Trust, Downs Road, Sutton, Surrey SM2 5NG, UK; j Clinical Trials and Statistics Unit, The Institute of Cancer Research, London SM2 5NG, UK; k Fundación Pública Galega de Medicina Xenómica, Servizo Galego de Saúde (SERGAS), 15706 Santiago de Compostela, Spain; l Department of Radiation Oncology, Tom Baker Cancer Center, University of Calgary, Calgary, Canada; m Cancer and Other Non-Infectious Diseases, MRC Clinical Trials Unit, London WC2B 6NH, UK; n Division of Radiation Oncology, Department of Oncology, Cross Cancer Institute, University of Alberta, Edmonton, Canada; o Department of Pathology, Albert Einstein College of Medicine, Bronx, NY, USA; p Department of Genetics, Albert Einstein College of Medicine, Bronx, NY, USA; q Department of Genetics and Genomics Sciences, Icahn School of Medicine at Mount Sinai, New York, NY, USA; r Department of Radiation Oncology, New York University School of Medicine, New York, NY, USA; s University of Cambridge, Department of Oncology, Cambridge Biomedical Campus, Addenbrooke's Hospital, Hills Road, Cambridge CB2 0QQ, UK; t Department of Oncology, Box 193, Cambridge University Hospitals NHS Foundation Trust, Hills Road, Cambridge CB0 0QQ, UK; u Institute of Cancer Sciences, The University of Manchester, Manchester Academic Health Science Centre, Christie Hospital, Manchester M20 4BX, UK

**Keywords:** SNP, single nucleotide polymorphism, GWAS, genome-wide association study, EBRT, external bean radiotherapy, BED, biologic effective dose, MAF, minor allele frequency, STAT, standardized total average toxicity, PCA, principle components analysis, TURP, transurethral resection of the prostate, LD, linkage disequilibrium, ENCODE, encyclopedia of DNA elements, eQTL, expression quantitative trait locus, GTEx, Genotype-Tissue Expression project, Radiogenomics, Genome-wide association study, Prostate cancer, Radiation toxicity, Cancer survivorship, Quality of life

## Abstract

Nearly 50% of cancer patients undergo radiotherapy. Late radiotherapy toxicity affects quality-of-life in long-term cancer survivors and risk of side-effects in a minority limits doses prescribed to the majority of patients. Development of a test predicting risk of toxicity could benefit many cancer patients. We aimed to meta-analyze individual level data from four genome-wide association studies from prostate cancer radiotherapy cohorts including 1564 men to identify genetic markers of toxicity. Prospectively assessed two-year toxicity endpoints (urinary frequency, decreased urine stream, rectal bleeding, overall toxicity) and single nucleotide polymorphism (SNP) associations were tested using multivariable regression, adjusting for clinical and patient-related risk factors. A fixed-effects meta-analysis identified two SNPs: rs17599026 on 5q31.2 with urinary frequency (odds ratio [OR] 3.12, 95% confidence interval [CI] 2.08–4.69, p-value 4.16 × 10^− 8^) and rs7720298 on 5p15.2 with decreased urine stream (OR 2.71, 95% CI 1.90–3.86, p-value = 3.21 × 10^− 8^). These SNPs lie within genes that are expressed in tissues adversely affected by pelvic radiotherapy including bladder, kidney, rectum and small intestine. The results show that heterogeneous radiotherapy cohorts can be combined to identify new moderate-penetrance genetic variants associated with radiotherapy toxicity. The work provides a basis for larger collaborative efforts to identify enough variants for a future test involving polygenic risk profiling.

## Highlights

• SNPs rs17599026 and rs7720298 increase risk of urinary toxicity following radiotherapy in prostate cancer patients.
• Data from heterogeneous radiotherapy cohorts can be meta-analyzed to identify genetic variants associated with toxicity.
• A SNP-based predictive assay could improve the therapeutic index of radiotherapy and aid in individualization of cancer treatment.

Risk of radiotherapy side-effects in a minority limits doses given to the majority. A test is needed to predict risks so treatments are personalized. Recent whole-genome studies in a few hundred patients found none or one genetic variant increasing side-effect risk. Larger studies are needed, but difficult because treatments differ between centers. Our study of > 1500 prostate cancer patients from four centers found two variants. The research shows combining heterogeneous radiotherapy datasets works and larger collaborative efforts identifying enough variants for a future test are worthwhile. As nearly 50% of cancer patients have radiotherapy, our work could benefit many people.

## 1 Introduction

Radiotherapy is used in the treatment of up to 50% of cancer patients and around 40% of long-term cancer survivors underwent radiotherapy at some point in their treatment. For example, approximately half of the 1.1 million men diagnosed with prostate cancer worldwide each year receive radiotherapy, and the 5-year relative survival rates approach 100% for non-metastatic disease (Howlader et al., 2013). Although modern treatments minimize radiation doses to surrounding normal tissues, some men develop long-term toxicity (Bentzen et al., 2010). The risk of severe toxicity limits doses, which aim to keep the prevalence below 5%. Mild and moderate effects are common (10–50% of those treated) (Alemozaffar et al., 2011, Dearnaley et al., 2012, Heemsbergen et al., 2006, Kneebone et al., 2004, Resnick et al., 2013, Syndikus et al., 2010), impact negatively on quality-of-life, and are an important factor when men consider treatment options (Davison et al., 2002).

There is a need for a test that reflects a cancer patient's radiosensitivity and predicts the likelihood of toxicity. Many assays have been explored but none proved sufficiently reliable for clinical application. Over the past 15 years interest increased in identifying the genetic variants associated with risk of toxicity. The rationale behind the work is that a future test based on a germline polygenic risk score will not suffer from the poor reproducibility associated with other assays measuring radiosensitivity (Barnett et al. 2015).

Mutations associated with well-characterized radiosensitivity syndromes such as ataxia telangiectasia (Taylor et al., 1975) are rare and do not explain the general inter-individual variation in toxicity following radiotherapy (Safwat et al., 2002). Rather, it is hypothesized that common genetic variants, such as single nucleotide polymorphisms (SNPs) account for most of the heritability of radiosensitivity (West and Barnett, 2011). Studies have begun to identify common variants associated with radiotherapy toxicity. Candidate gene studies showed rs2868371 in *HSPB1* (MIM 602195) (Lopez Guerra et al., 2011, Pang et al., 2013) and rs1800469 in *TGFB1* (MIM 190180) (Guerra et al., 2012) are associated with late effects of lung radiotherapy; and rs1800629 in *TNF* (MIM 191160) (Talbot et al., 2012) and rs1139793 in *TXNRD2* (MIM 606448) (Edvardsen et al., 2013) are risk SNPs for late toxicity following breast radiotherapy. Genome-wide association studies (GWAS) identified a locus on chr11q14.3 associated with rectal bleeding (Kerns et al., 2013b) and a locus on chr2q24.1 within *TANC1* (MIM 611397) associated with overall toxicity (Fachal et al., 2014) following radiotherapy for prostate cancer. Another study showed more associations at the p-value < 5 × 10^− 7^ level than expected by chance, providing the strongest evidence to date that many common genetic variants are associated with risk of toxicity (Barnett et al., 2014).

Recently published GWAS have limitations that we aimed to overcome by using a meta-analysis approach. The published studies used a multi-stage approach, where a small first-stage cohort was analyzed for a genome-wide panel of SNPs and only the most significant SNPs were genotyped in validation datasets. Thus, true positive SNPs were likely missed because they were not tested in the full set of individuals. Here, the Radiogenomics Consortium (West and Rosenstein, 2010) undertook a meta-analysis of four GWAS in order to maximize statistical power (Cohn and Becker, 2003) to discover additional risk variants. It is known that risk factors for late toxicity include not only genetics but also dosimetric parameters, co-morbidities, and patient demographics (Barnett et al. 2009). The latter factors can vary between cohorts as can the treatment (e.g. in prostate cancer: external beam or brachytherapy; type of fractionation – large or small doses per fraction; variable use of hormone therapy; variable use of surgery) and scales used to assess toxicity. There were concerns, therefore, whether the heterogeneity across cohorts might limit our ability to identify variants.

This study is important because our ability to identify enough SNPs for a risk profile for clinical implementation is dependent on combining multiple heterogeneous cohorts. The aim was to show that multi-center radiotherapy cohorts could be harmonized and analyzed to identify risk SNPs by increasing the number of individuals analyzed in a single stage (Skol et al., 2006). STROGAR guidelines (Kerns et al., 2014) for reporting radiogenomic studies, which build on the STREGA and STROBE guidelines (Little et al., 2009, von Elm et al., 2007), were followed throughout.

## 2 Subjects & Methods

### 2.1 Participants

The four cohorts (RAPPER, RADIOGEN, Gene-PARE, and CCI) comprised individuals with adenocarcinoma of the prostate treated with radiotherapy with curative intent. Table 1 shows the number of individuals in each cohort the number with genome-wide SNP data available, and the final number included in the GWAS meta-analysis after excluding samples for quality control or due to missing data. Informed consent was obtained from all study participants and all studies conform to standards indicated by the Declaration of Helsinki.

RAPPER (UKCRN1471; n = 727) (Burnet et al., 2006) was approved by the Cambridge South Research Ethics Committee (05/Q0108/365). Individuals received neoadjuvant androgen suppression and external beam radiotherapy, (EBRT): 233 from MRC RT01 (ISRCTN47772397) (Sydes et al., 2004) and 494 from CHHiP (ISRCTN97182923) (Dearnaley et al., 2012).

RADIOGEN (n = 741) was approved by the Galician Ethical Committee. Individuals received conformal radical or post-prostatectomy EBRT at the Clinical University Hospital of Santiago de Compostela, Spain (Fachal et al., 2012), and 511 individuals had hormone therapy.

Gene-PARE (n = 895) (Ho et al., 2006) was approved by the Mount Sinai Medical Center Institutional Review Board. Individuals had brachytherapy with/without EBRT at the Mount Sinai Hospital, New York, and 472 received hormone therapy. ^125^I (160Gy; TG-43) was used in those undergoing brachytherapy alone and ^103^Pd (124Gy) in those also receiving EBRT (Stock et al., 1995, Stone et al., 2003).

The CCI cohort (n = 155) (Kerns et al., 2013b) was approved by the Health Research Ethics Board of Alberta (Cancer). Individuals were recruited from the Cross Cancer Institute in Edmonton and the Tom Baker Cancer Centre in Calgary, Alberta, Canada. All 155 individuals underwent EBRT and 82 received hormonal therapy.

Total biologic effective dose (BED) (Stock et al., 2006) was calculated for individuals in all four studies to compare radiation exposure across studies (α/β = 3).

### 2.2 Assessment of Late Radiotherapy Toxicity

Participants were assessed prospectively for toxicity (see Table S1). Toxicity was measured 1.5–2.5 years following treatment with the latest value used if more than one assessment was recorded during this follow-up window. For rectal bleeding in Gene-PARE, a 1–5 year window was allowed, because the scoring system assigns grades based on whether rectal bleeding occurs as a single incident or intermittent symptoms over time. Urinary endpoints were re-graded to harmonize across the studies (Table S2). As urinary daytime frequency and nocturia were recorded separately (RAPPER, Gene-PARE) or as a single endpoint (RADIOGEN, CCI), they were collapsed into a single endpoint in RAPPER and Gene-PARE by taking the maximum score between the two.

Changes in scores from baseline were calculated for each endpoint. Improvement from baseline (i.e. a negative change in score) was coded as 0. Table S3 lists the two-year prevalence of the toxicity endpoints in the four cohorts, including samples that were not genotyped. A Standardized Total Average Toxicity (STAT) score was calculated as described previously (Barnett et al., 2012b) to provide an overall measure of toxicity. All samples with 2-year toxicity data available and the endpoints described in Table S1 were used to calculate STAT.

Data simulation determined the most statistically powerful way to analyze toxicity: ordinal logistic regression of exact change in toxicity grade from baseline; binary logistic regression with no change in toxicity grade compared with a ≥ 1 point increase in toxicity grade; or binary logistic regression with change in toxicity grade ≤ 1 point compared with a ≥ 2 point change. Genotype and toxicity phenotype data for 600 individuals were simulated under two models: (1) null (no association), and (2) alternative model of linear association characterized by a log-linear per-allele increase in odds of toxicity. Under both types of model, genotypes were randomly assigned to each subject according to one of five minor allele frequencies (MAF): 0.05, 0.15, 0.25, 0.35 or 0.45. To create the null data set, a toxicity grade was also randomly assigned to each subject. Datasets representing a log-linear association between genotype and toxicity were created by assigning each subject a toxicity risk score that was calculated using their genotype and a pre-specified effect size (log(odds ratio) = log(1.5)):

$$R_{i}=\left({\text{genotype}}_{i}\times \beta \right)-\left(2\times \mathrm{MAF}\times \beta \right)$$

for subjects i = 1 … n;

β the pre-specified effect size;

(2 × MAF × β) the population average.

A random risk was also added to each score, representing non-genetic factors that may influence toxicity, to create a logistic distribution of toxicity risk. This logistic distribution was then used to assign a toxicity grade to each subject such that subjects lying at the high end of the risk distribution had higher toxicity grades than those at the low end of the distribution. Under both types of model, two phenotype distributions were simulated to reflect the distributions of urinary frequency and rectal bleeding observed in RAPPER.

Each simulation was repeated 100,000 times. Three regression models were fitted to each set of simulated data, with toxicity grade as the outcome variable and genotype as the independent variable. Each model produced a p-value for comparison.

The power of each type of model applied to the log-linear association data was assessed by tabulating the number of p-values (out of 100,000) achieving statistical significance. Applying a simple Bonferroni correction to the observed p-values to account for 100,000 test repeats, a test was considered statistically significant if p-value < 5 × 10^− 6^. The model that detected the most significant associations was deemed most powerful. p-Values obtained from analyses of the null data sets were assessed for type I error.

Results from 100,000 simulations showed that both ordinal logistic and binary (no versus ≥ 1 point change) logistic regression were equally powerful statistical approaches (Table S4), and binary logistic regression (no change in toxicity grade compared with a ≥ 1 point increase in toxicity grade) was used for the primary analysis. No model displayed higher-than-expected type I errors. Data simulation was performed using R (R Core Development Team, 2014) with the Ordinal package (Christensen, 2013).

### 2.3 Genotyping, Quality Control and Imputation

Germline DNA was genotyped using commercial SNP arrays as part of previously completed GWAS (Barnett et al., 2014, Fachal et al., 2014, Kerns et al., 2013a, Kerns et al., 2013b, Kerns et al., 2013c). Quality control filtering removed SNPs that were missing in > 2.5% of samples, had MAF < 1% or displayed frequencies deviating from those expected under Hardy-Weinberg Equilibrium (p-value < 10^− 6^). Samples with > 5% of SNPs missing, showing cryptic relatedness, showing excess heterozygosity or being principle component analysis (PCA) outliers were removed (Table 1). After filtering, the four datasets had genotyping rates > 99%. To minimize potential confounding by population structure, individuals with non-European ancestry (Table 1) were excluded using PCA performed with samples of known ancestry from the International HapMap Project (International HapMap, 2003).

Imputation using IMPUTE2 software (Howie et al., 2009) with the 1000 Genomes phase I, version 3 (release date 3/16/2012) reference panel (1092 individuals from all 14 populations) (Genomes Project et al., 2012) yielded ~ 38 million SNPs, small insertions and deletions, and structural variants in each study. These datasets were filtered as above in addition to removing SNPs with information score, a measure of imputation certainty, < 0.3. The final datasets included in the GWAS meta-analysis were: 6,672,177 SNPs in 527 RAPPER participants; 6,767,156 SNPs in 597 RADIOGEN participants; 6,627,946 SNPs in 290 Gene-PARE participants; and 6,504,337 SNPs in 150 CCI participants.

SNP rs17599026 was directly genotyped in the RADIOGEN and RAPPER samples using a TaqMan assay (forward primer sequence TGCTCATGATGAAGGTATGCTTTCT, reverse primer sequence ACAAAACTGTATTCCCAAGACAAAGC and probe sequences CACCATCCTAAAGCAGTG plus ACCATCCTAAAACAGTG; Applied Biosystems, Thermo Fisher Scientific) according to the manufacturer's standard protocol (PCR annealing and extension performed at 60 °C for 1 min × 40 cycles). It was not possible to design a TaqMan assay with high specificity for rs7720298 as it is immediately adjacent to another SNP (rs7720176).

### 2.4 Statistical Analysis

Individual GWAS were analyzed using binary logistic (individual toxicity endpoints) or linear (STAT) regression, adjusting for non-genetic risk factors identified by QUANTEC (Michalski et al., 2010, Viswanathan et al., 2010). These were: age at treatment, diabetes, rectal volume and BED in analysis of rectal bleeding; age at treatment, transurethral resection of the prostate (TURP) prior to radiotherapy, baseline symptom grade and BED in analysis of urinary endpoints; and all factors in analysis of STAT. Hormone therapy was also included in the Gene-PARE and CCI analyses because baseline symptoms were assessed prior to completion of hormone therapy. Multivariable regression was performed using SNPTEST software (Marchini et al., 2007) with the frequentist test and expected method. The expected method uses the genotype dosages produced from imputation, which account for the uncertainty in using imputed genotypes rather than experimentally determined genotypes. Thus, inaccuracy related to using imputed data was accounted for in the statistical analysis. An additive genetic inheritance model was assumed in all analyses.

A fixed-effects meta-analysis with inverse variance weighting was performed using the regression beta coefficients and standard errors produced by SNPTEST. Studies have shown fixed-effects to be more statistically powerful compared with random-effects when the primary purpose is SNP discovery rather than refinement of the effects size estimate (Evangelou and Ioannidis, 2013). A chi-squared test of heterogeneity was performed for each SNP. SNPs considered significant had a meta-p-value ≤ 5 × 10^− 8^ and agreement in effect direction across all cohorts. Significant SNPs were re-analyzed using an ordinal logistic regression model.

Regions of linkage disequilibrium were defined based on r^2^ ≥ 0.5 in SNPs from the 1000 Genomes European population with the meta-analysis top hits using Haploview software (Barrett et al., 2005). The most recent release of the 1000 Genomes data (version 5, release date 5/2/2013) was used to estimate linkage disequilibrium.

### 2.5 Power Calculations

Power calculations were performed using the web-based Genetic Power Calculator (Purcell et al., 2003) assuming a sample size of 1564 individuals, a phenotype prevalence of 20%, and type I error of 5 × 10^− 8^.

## 3 Results

Table 2 summarizes the cohort characteristics and Table 3 the toxicity prevalences of the 1564 men treated for prostate cancer who were included in the analysis. Two years following radiotherapy 17.8% (277 of 1557) of individuals experienced rectal bleeding, 15.0% (212 of 1410) an increase in urinary frequency, and 8.1% (101 of 1245) a decrease in urine stream. The meta-analysis had ≥ 99% power to detect SNPs with MAF ≥ 10% and per-allele effect size ≥ 2 assuming a genome-wide significance threshold of 5 × 10^− 8^ (Table 4).

**Fig. 1:**
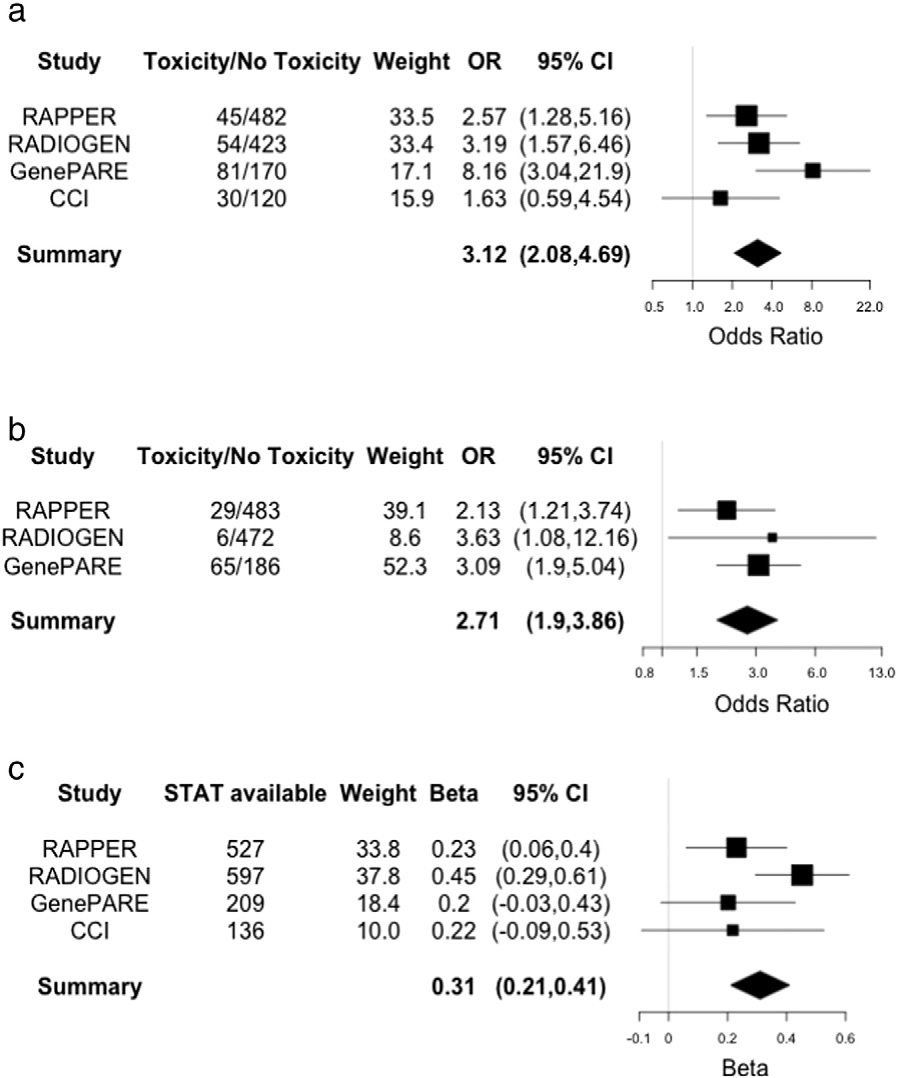
Forest plots of significant SNPs. SNPs with meta-p-value < 5 × 10^− 8^ associated with: (a) urinary frequency (rs17599026); (b) decreased urine stream (rs7720298); and (c) overall toxicity (rs11230328). p-Values are from meta-analysis of regression coefficients and standard errors from regression analysis performed in each study. Odds ratios (OR; urinary frequency and decreased urine stream) or regression beta coefficient (STAT score) are shown for each individual study as well as the meta-analysis (Summary). The size of the box marking each odds ratio or beta is proportional to the precision of estimate for the given study. Lines on the boxes denote 95% confidence intervals. ‘Toxicity’ and ‘No toxicity’ (rs17599026 and rs7720298) was defined as ≥ 1 point increase in toxicity grade versus no change in toxicity grade from pre-radiotherapy scores. Overall toxicity was analyzed as a continuous variable (STAT).

Meta-analysis Q-Q plots (Fig. S1) show no genomic inflation, suggesting that population structure was adequately controlled using principal components analysis to exclude outliers with non-European ancestry. The most statistically significant SNPs associated with late toxicity are listed in Table 5, Table 6, Table 7, Table 8, which show several SNPs had meta-p-values reaching or approaching significance (p-value ≤ 5 × 10^− 6^) with concordant effect direction across the individual studies. Three SNPs reached genome-wide significance: rs17599026 on 5q31.2 with urinary frequency (3.12, 95% CI 2.08–4.69, p-value 4.16 × 10^− 8^); rs7720298 on 5p15.2 with decreased urine stream (OR 2.71, 95% CI 1.90–3.86, p-value = 3.21 × 10^− 8^); and rs11230328 on 11q12.2 with STAT score (Beta 0.31, 95% CI 0.21–0.41, p-value = 6.27 × 10^− 10^). rs17599026 was directly genotyped in the GenePARE and CCI studies and imputed in the RADIOGEN (information score = 0.90) and RAPPER (information score = 0.87) studies. rs7720298 was imputed in all four studies (RADIOGEN information score = 0.91, RAPPER information score = 0.93, GenePARE information score = 0.94, and CCI information score = 0.95) and rs11230328 was imputed in all four studies (RADIOGEN information score = 0.68, RAPPER information score = 0.61, GenePARE information score = 0.88, and CCI information score = 0.97). To provide a more interpretable effect size for rs11230328, STAT score was dichotomized at the mean and analyzed using logistic regression, which resulted in an OR of 1.59 (95% CI 1.25–2.02). There were no genome-wide significant SNPs associated with rectal bleeding, and though several approached significance, these require investigation in future larger studies. Fig. 1 shows forest plots for the SNPs that reached the genome-wide significance threshold.

In order to confirm the quality of the imputation, rs17599026 was genotyped directly in the RADIOGEN and RAPPER samples. Genotyping was successful in 645 of 654 RADIOGEN samples (correlation between the imputed and genotyped data of 0.88) and 595 of 600 RAPPER samples (correlation between the imputed and genotyped data of 0.85). These results confirmed that the genotypes are in strong agreement with the imputed data. When the direct genotype data was used in analysis, rs17599026 showed an association signal consistent with that seen using the imputed data, though the p-value fell short of the strict threshold for genome-wide significance (OR 2.48, 95% CI 1.68–3.63, p-value = 3.66 × 10^− 6^).

Associations between the top SNPs (meta-analysis p-value ≤ 5 × 10^− 6^) and toxicity severity were modeled using ordinal logistic regression (Table 9). For all SNPs, the p-value from ordinal logistic regression was similar to that from binary logistic regression. For several SNPs the association signal improved slightly with the ordinal logistic model: rs12497518, rs141044160, rs6999859, and rs4804134 associated with rectal bleeding; rs17599026, rs7366282, rs4534636, rs10101158, rs10209697, rs6003982, and rs7356945 associated with urinary frequency; rs7720298, rs673783, rs62091368, and rs144596911 associated with decreased urine stream.

**Fig. 2:**
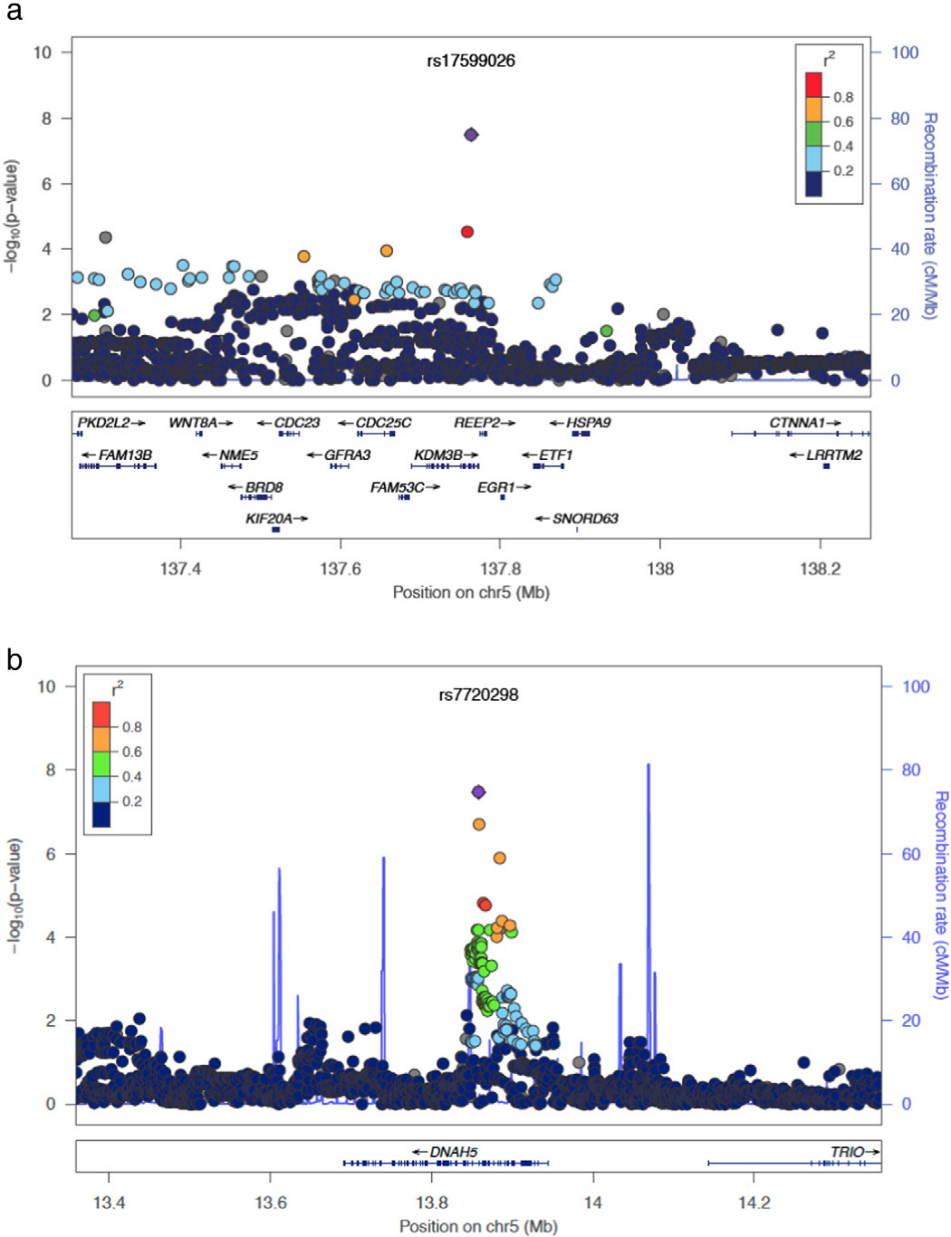
Regional plots for two loci. Loci tagged by rs17599026 (a) and rs7720298 (b) with points representing the tag SNP reported in this paper shown in purple circles. Points representing nearby SNPs are color coded according to linkage disequilibrium r^2^ value as indicated in the legends. The recombination rate is estimated from samples from the International HapMap project.

Regions of linkage disequilibrium (LD) were defined for the three loci tagged by SNPs reaching genome-wide significance. rs17599026, associated with urinary frequency, tags a 106 kb region of LD (base position 137,657,783–137,763,798; Fig. 2A) containing part of *KDM3B* (including the promoter region through exon 20), the upstream *FAM53C*, and part of the upstream *CDC25C* (the promoter region through exon 6). rs7720298, associated with decreased urine stream, tags a 39 kb region of LD (base position 13,858,328–13,897,362; Fig. 2B) that contains exons 16 through 30 of *DNAH5*. rs11230328 was not in strong linkage disequilibrium with any other common SNPs found in the more recent release of the 1000 Genomes population data, and this locus may represent a spurious association. rs11230328 lies within a LINE element, and may be difficult to map in the genome due to its location within a region of high homology. Imputation coverage (i.e. the number of SNPs successfully imputed in the study datasets out of common SNPs (MAF ≥ 0.05) within the 1000 Genomes European population) was high within the regions tagged (correlation r2 ≥ 0.5) by rs17599026 (173/183, 94.5%) and rs7720298 (130/136, 95.6%).

SNPs rs17599026 and rs7720298 are located in non-coding regions, as is common in GWAS, and the LD blocks tagged by these SNPs cover coding and non-coding regions. rs17599026, associated with urinary frequency, lies in an intronic region 23 bp downstream of exon 20 of *KDM3B* (MIM 609373; NM_016604.3:c4753 + 23C > T) encoding the lysine-specific demethylase 3B protein. *KDM3B* is highly expressed in bladder tissue from the Human Protein Atlas project (Uhlen et al., 2015), suggesting that this gene could be involved in normal bladder function and potentially dysfunction following damage from radiation exposure. rs17599026 itself does not lie in a site of known transcription factor binding or chromatin modification from the Encyclopedia of DNA Elements (ENCODE) catalog (ENCODE Project Consortium, 2012) and there are no significant expression quantitative trail loci (eQTLs) for this SNP in the Genotype-Tissue Expression (GTEx) project (GTEx Consortium, 2013). Given that rs17599026 is very close to exon 20, it could have an effect on splicing. However, no significant splicing motif alteration was detected using Human Splicing Finder (Desmet et al., 2009). ENCODE data show that the large LD block tagged by this SNP contains multiple transcription factor binding sites, DNase hypersensitive sites, histone methylation sites (methylation of lysine 4 at histone 3, H3K4Me3 and methylation of lysine 4 at histone 1, H3K4Me1), and histone acetylation sites (acetylation of lysine 27 at histone 3, H3K27Ac) that may affect regulation of the nearby genes. rs7720298, associated with decreased urine stream, lies in an intronic region just downstream of exon 30 of *DNAH5* (MIM 603335; NM_001369.2:c.4950 + 1233G > C) encoding the dynein, axonemal, heavy chain 5 protein that is part of a microtubule-associated motor protein complex. Rare mutations in *DNAH5* can result in development of abnormal cilia and flagella in cells that lead to primary ciliary dyskinesia, which is a disorder characterized in part by chronic respiratory tract infections (Escudier et al., 2009). In addition to playing an important role in the lung, *DNAH5* is expressed in both kidney and bladder tissue, suggesting a biologic role in normal function of the urinary tract (Uhlen et al., 2015). This SNP does not lie in a site of known transcription factor binding or chromatin modification from the ENCODE catalog nor is it an eQTL based on data from GTEx, but the region of LD tagged by rs7720298 contains several transcription factor binding sites and sites of DNase hypersensitivity measured in ENCODE cell lines, suggesting that it may tag a site of transcriptional regulation.

## 4 Discussion & Conclusions

This meta-analysis aimed to identify SNPs associated with late radiotherapy toxicity in a single tumor site. By having a total sample size in a single stage analysis of > 1500 men with prostate cancer (versus ~ 600 in published studies), the study identified two risk loci. This study had ≥ 99% power to identify common SNPs (MAFs ≥ 10%) that confer a relatively large increased risk for developing late toxicity (OR ≥ 2.0). Identification of multiple loci in this single-stage meta-analysis versus single loci in published GWAS that used a staged approach is consistent with the increased power. The meta-analysis showed that heterogeneity in radiotherapy datasets is not a barrier for future multi-cohort radiogenomic studies. The absence of significant SNPs associated with rectal bleeding is consistent with the relatively limited statistical power. Most common variants identified via GWAS have more modest effects (ORs 1.15 to 1.5) than this study was powered to detect. Our group previously reported an excess of associations at the p < 5 × 10^− 7^ level for rectal bleeding showing many SNPs should be identified as sample sizes increase (Barnett et al., 2014). In addition, our current approach of dichotomizing toxicity at grade 0 versus grade 1 or worse and considering two years of follow-up may not be optimal for rectal bleeding, which could be explored as our radiogenomic cohorts increase.

The significant SNPs lie in non-coding regions, as is common in GWAS where ~ 64% of identified SNPs lie in enhancer regions (Hnisz et al., 2013). Gene enhancer elements are noncoding segments of DNA involved in regulating transcriptional programs (Corradin and Scacheri, 2014). Their location is predicted by DNase I hypersensitive regions of open chromatin flanked by sties of histone methylation. These enhancers can be active (associated with the acylation of lysine 27 of histone 3 [H3K27Ac]) or repressed (correlated with histone marks H3K27Me3 and H3K9Me3). Although premature to speculate on the functional consequences of these SNPs, they lie in intronic regions of genes that are expressed in tissues exposed to radiation during treatment of prostate cancer and are involved in the symptoms experienced by men having radiotherapy for this malignancy. Fine mapping studies and functional characterization of causal variants contained within these loci should expand our understanding of the biology underlying the pathogenesis of radiotherapy toxicity.

rs17599026 was genotyped to check the quality of the imputed data from RADIOGEN and RAPPER. Direct genotyping confirmed the high quality of the imputation, but we were unable to genotype directly the other two SNPs for which imputed data were used for all four cohorts. rs7720298 had high imputation information scores (> 0.95) in all three studies of urinary decreased stream, lending confidence to this association. However, rs11230328 had a lower imputation quality (< 0.70) in two of the four studies of overall toxicity, and it is not present in the latest 1000 Genomes Project release (v5a; 05/02/2013). Thus, rs11230328 may represent a spurious association.

None of the previously studied candidate gene SNPs for radiotherapy toxicity were among the top loci identified in this meta-analysis, consistent with a recent validation study showing no evidence for association between 92 candidate SNPs and radiotherapy toxicity among ~ 1600 individuals treated with radiotherapy for breast or prostate cancer (Barnett et al., 2012a). No SNP discovered in previous GWAS was identified in this de novo analysis, likely due to methodological differences. The published locus on chr2q24.1, identified in RADIOGEN and replicated in RAPPER and Gene-PARE (Fachal et al., 2014), was excluded because the MAFs of the tagging SNPs were < 5%, the threshold set in this meta-analysis to maximize statistical power. Subsequent to the meta-analysis, we tested SNPs in the chr2q24.1 locus for replication in CCI (the only cohort not included in the initial publication) using the methods of the originally published paper. All seven previously reported SNPs showed a consistent direction of association with overall toxicity in CCI, and rs264651 showed a statistically significant association (linear regression beta = 0.66; 95% CI 0.06, 1.27; p-value = 0.03). The locus was also tested for association with the three individual toxicity endpoints investigated in this paper, and appeared to be predominantly associated with urinary toxicity (Table S5). SNPs rs7582141, rs6432512, rs264588, and rs264631 were significantly associated with an increased risk for decreased urinary stream and increased urinary frequency (meta-analysis p-values < 0.05, Table S5). The directions of the associations were consistent across the other three SNPs, with rs264663 and rs264651 reaching statistical significance for urinary frequency. The direction of the association of six of the seven SNPs was consistent for the rectal bleeding endpoint, but none of the SNPs approached statistical significance, suggesting that the urinary endpoints are predominantly driving the association of this locus with overall toxicity. This finding thus supports the initial report that chr2q24.1 is a risk locus for late radiotherapy toxicity in prostate cancer. rs7120482 on chr11q14.3 was previously identified in Gene-PARE and replicated in a combined dataset that included RADIOGEN and CCI (Kerns et al., 2013b), but the association with rectal bleeding was restricted to a recessive inheritance model. In this meta-analysis, dominant and recessive models were not considered in order to avoid an increased burden of multiple comparisons correction. Subsequent to this meta-analysis, we tested rs7120482 for replication in RAPPER (the only cohort not included in that initial publication) using the methods of the originally published paper. The initial association with rectal bleeding was not replicated in RAPPER (odds ratio = 0.74; 95% CI 0.08, 7.33; p-value = 0.79). This lack of replication could be due to differences in the radiotherapy regimen or scoring of toxicity - the incidence of rectal bleeding was low in RAPPER (3%) compared with the other studies (10% to 18%). However, the initial association fell just short of genome-wide significance and might be a false positive result. At this early stage of radiogenomic GWAS we expect some initial associations to be lost as sample sizes increase and false-positive findings are reduced, as seen in other GWAS meta-analyses (Evangelou and Ioannidis, 2013). Finally, Gene-PARE previously identified risk SNPs for erectile dysfunction (Kerns et al., 2010, Kerns et al., 2013a), but this endpoint was not assessed in the other studies in the meta-analysis. Thus, previously identified SNPs remain important, and the meta-analysis reports additional loci.

This study has several strengths. First, toxicity was assessed prospectively in all four studies, which minimizes recall bias. Second, baseline information ensures any SNP-toxicity associations identified occur because of the radiotherapy rather than any pre-existing (possibly tumor-related) symptoms. Third, a multivariable approach accounted for relevant treatment and clinical variables. Radiogenomics involves phenotypes that occur in response to an environmental exposure (i.e. therapeutic radiation) that is measurable and can be adjusted for. Fourth, reliable and current imputation methods were used to obtain comparable and dense SNP maps across the four studies, allowing meta-analysis of studies involving different SNP genotyping platforms. Without imputation, only 15,144 SNPs were common to all four datasets, reducing the likelihood of identifying SNPs associated with late radiotherapy toxicity.

This study has limitations. First, the four studies were designed independently and involved different methods for recording toxicity. Endpoints needed harmonizing and the approach might be sub-optimal, but serves as a useful guide for future radiogenomics studies involving multiple cohorts. Although endpoints were dichotomized to minimize differences in toxicity grades across the cohorts, the simulation experiment showed minimal loss of statistical power. Second, there was the heterogeneity in radiotherapy protocols. For example, Gene-PARE included individuals who received brachytherapy with EBRT, which increases urinary toxicity (Sanda et al., 2008). Similarly, the proportion of individuals who received androgen deprivation differed across the studies. This heterogeneity was adjusted for using multivariable analysis and by a meta-analysis approach. Importantly, none of the top SNPs showed evidence of heterogeneity in effect size across studies (Table 5, Table 6, Table 7, Table 8). The first SNPs identified are those that rise above such noise in the data, and the significance of our work is that we can find variants despite imperfect datasets. We attempted to control for differences in radiation dose by adjusting for total biologically effective dose in each GWAS. However, this is a surrogate for the doses received by specific normal tissues. Future cohorts with detailed dosimetry data to the different organs at risk will improve on our ability to identify SNPs. The heterogeneity in cohorts should be embraced, as any predictive test must use SNPs that are associated with toxicity across treatment centers and protocols. Last, in order to minimize confounding by population structure, the present analysis was restricted to individuals of European ancestry. It will be important for future studies to focus on other ancestral groups, both for replication of the loci identified here and for discovery of additional loci.

While this study was successful in identifying radiosensitivity SNPs via a meta-analysis of GWAS, it is modestly sized compared with GWAS of other diseases and traits, and it should be viewed as the starting point for expanded radiogenomics studies. Given that our study was not powered to detect SNPs with odds ratio < 2, many true positive SNPs will have been missed. Also, though the SNPs identified here reach statistical significance and show consistency across multiple independent studies, there is still a possibility that they will fail to replicate in other cohorts. This is a challenge in GWAS (McCarthy et al., 2008), but meta-analysis and replication studies have proved successful in validating hundreds of risk SNPs for a wide variety of diseases and phenotypes. Future large GWAS meta-analyses will identify additional SNPs, and this work is underway. The Radiogenomics Consortium is a participant in the OncoArray Network, which is a large collaborative effort to gain new insight into the genetic architecture and mechanisms underlying several cancers as well as outcomes related to the their treatment. Approximately 5000 additional DNA samples from individuals treated with radiotherapy for prostate cancer are being genotyped using the customized OncoArray, and meta-analysis of this greatly expanded set of GWAS data using the methods developed in this paper will uncover additional risk SNPs, provide a platform for further validation of the SNPs identified here, and serve as the basis for future post-GWAS analyses on these confirmed loci.

The last decade saw a rapid expansion of knowledge of the genetics of disease susceptibility in the general population. Numerous large collaborative GWAS showed that polygenic risk profiles can be built based on multiple SNPs each conferring small effects but together a significant proportion of susceptibility to common diseases. With the rapid decline in costs for genetic testing, there is growing acceptance that risk prediction models incorporating genetic and environmental factors will be important in future healthcare provision (Chatterjee et al., 2016). In 2012 there were an estimated 32.6 million people alive five years after being diagnosed with cancer (http://www.cdc.gov/cancer/international/statistics.htm), and many will be living with the consequences of treatment. Research developing models predicting susceptibility to long-term radiation effects is important and the findings reported here shows that heterogeneous radiotherapy cohorts can be combined to identify common genetic variants associated with toxicity. The work provides a basis for larger collaborative efforts to identify enough variants for a future test involving polygenic risk profiling.

## Funding Sources

This work was supported by Cancer Research UK (C1094/A11728 to CMLW and NGB for the RAPPER study, C26900/A8740 to GCB, and C8197/A10865 to AMD), the Royal College of Radiologists (C26900/A8740 to GCB), the National Institute for Health Research (GCB; no grant number), Addenbrooke's Charitable Trust (GCB; no grant number), Institute of Cancer Research (National Institute for Health Research) Biomedical Research Centre (C46/A2131 to DPD and SG), the National Institute for Health Research Cambridge Biomedical Research Centre (NGB; no grant number), UK Medical Research Council (RG70550 to LD), the Joseph Mitchell Trust (AMD; no grant number), the Experimental Cancer Medicine Centre (CMLW; no grant number), Cancer Research UK Program grant Section of Radiotherapy (C33589/A19727 to SLG), the United States National Institutes of Health (1R01CA134444 to BSR and HO, 2P30CA014520-34 to SB, and 1K07CA187546-01A1 to SLK), the American Cancer Society (RSGT-05-200-01-CCE to BSR), the U.S. Department of Defense (PC074201 to BSR and HO), Mount Sinai Tisch Cancer Institute Developmental Fund Award (BSR; no grant number), the Instituto de Salud Carlos III (FIS PI10/00164 and PI13/02030 to AV and PI13/01136 to AC), Fondo Europeo de Desarrollo Regional (FEDER 2007–2013 to AV and AC; no grant number), Instituto de Salud Carlos III (FIS PI10/00164 and PI13/02030 to AV and PI13/01136 to AC), Xunta de Galicia and the European Social Fund (POS-A/2013/034 to LF), and the Alberta Cancer Board Research Initiative Program (103.0393.71760001404 to MP). AMD receives support from the REQUITE study, which is funded by the European Union's Seventh Framework Programme for research, technological development and demonstration under grant agreement no. 601826. Laboratory infrastructure for the RAPPER study was funded by Cancer Research UK [C8197/A10123] and the Manchester Experimental Cancer Medicine Centre. The RAPPER cohort comprises individuals and data recruited into the RT01 and CHHiP UK radiotherapy trials. The RT01 trial was supported by the UK Medical Research Council. The CHHiP trial (CRUK/06/016) was supported by the Department of Health and Cancer Research UK (C8262/A7253); trial recruitment was facilitated within centers by the National Institute for Health Research Cancer Research Network. DPD and SLG acknowledge NHS funding to the NIHR Biomedical Research Centre at the Royal Marsden NHS Foundation Trust and Institute of Cancer Research.

## Conflicts of Interest

The authors declare no conflicts of interest. No one was paid to write this article by a pharmaceutical company or agency.

## Web Resources

Online Mendelian Inheritance in Man (http://www.omim.org)

Genetic Power Calculator (http://pngu.mgh.harvard.edu/~purcell/gpc/)

R statistical computing environment (http://www.R-project.org)

Centers for Disease Control and Prevention, Global Cancer Statistics (http://www.cdc.gov/cancer/international/statistics.htm)

## Author Contributions

SLK, LD, LF and GCB analyzed and interpreted data and drafted the initial manuscript. DPD, SLG, EH, MRS, NGB, GCB and CMLW collected data for the RAPPER study. NGB and CMLW were the principal investigators for the RAPPER study. AMC, AC, AGC, PP and AV collected data for the RADIOGEN study. AV was the principal investigator for the RADIOGEN study. RS, NNS, SLK, HO and BSR collected data for the Gene-PARE study. HO and BSR were the principal investigators for the Gene-PARE study. MS, and MP collected data for the CCI study. MP was the principal investigator for the CCI study. SA designed and performed TaqMan genotyping assays. SLK, LD, LF, SB, PDPP, DRB, KM, JPT, HO, AMD, GCB and CMLW contributed to the data analysis plan and statistical methods used. All authors contributed intellectual input to the study design and interpretation of results, and all authors reviewed the manuscript prior to submission. CMLW approved the final manuscript for submission.

## Figures and Tables

**Table 1:** Number of individuals in each cohort.

	RAPPER	RADIOGEN	Gene-PARE	CCI
Total in cohort	727	741	895	155
Genotyped via genome-wide SNP chip	672	741	381	155
Excluded: > 5% SNPs missing	18	1	8	1
Excluded: cryptic relatedness	14	19	16	0
Excluded: excess heterozygosity	19	–	–	–
Excluded: PCA outlier	–	68	–	–
Excluded: non-European ancestry based on PCA w/ HapMap samples	21	1	67	3
Excluded: lacking all 2 yr toxicity or essential covariate data	73	55	0	1
Number included in analysis of at least one toxicity endpoint	527	597^a^	290^b^	150^c^

a Additional follow-up of the RADIOGEN cohort increased the number with late toxicity data available from the 417 reported previously (Fachal et al., 2014). Of the 597 participants in RADIOGEN, one lacked data on rectal toxicity and seven data on rectal volume and were excluded from the analysis of rectal bleeding. 120 participants had no data on baseline urinary frequency and 119 were missing data on decreased urine stream and were excluded from the respective analyses.

b Of the 290 participants in Gene-PARE, 55 were lacking data for rectal volume and were excluded from analysis of rectal bleeding. 35 participants did not complete the urinary questionnaire and were excluded from analysis of urinary frequency and decreased urine stream.

c Of the 150 participants in CCI, 15 were lacking data for rectal volume or diabetes and were excluded from analysis of rectal bleeding.

**Table 2:** Individual study patient characteristics.

	RAPPER, N = 527	RADIOGEN, N = 597	Gene-PARE, N = 290	CCI, N = 151
Age, mean (sd^a^)	67 (5.7)	71 (5.8)	64 (7.5)	67 (7.2)
TURP^b^, n (%)	56 (11%)	45 (8%)	6 (2%)	6 (4%)
Diabetes, n (%)	39 (7%)	144 (24%)	16 (6%)	24 (16%)
Gleason score, n (%)				
≤ 6	261 (50%)	366 (61%)	193 (67%)	30 (20%)
7	231 (44%)	164 (28%)	69 (24%)	97 (64%)
≥ 8	25 (5%)	60 (10%)	28 (10%)	21 (14%)
missing	10 (2%)	7 (1%)	0	3 (2.0%)
Stage, n (%)				
T1	187 (35%)	214 (35.8%)	150 (52%)	38 (25%)
T2	267 (51%)	323 (54.1%)	128 (44%)	89 (59%)
T3	61 (10%)	44 (7.4%)	12 (4%)	16 (11%)
T4	0	0	0	1 (1%)
missing	12 (2%)	17 (2.8%)	0	7 (5%)
Pre-treatment PSA^c^, mean (sd)	13.2 (7.5)	16 (20.5)	9.4 (19.4)	15.5 (13.4)
Hormone therapy, n (%)	527 (100%)	415 (69.5)	147 (51%)	74 (49%)
BED^d^, mean (sd)	120.5 (6.2)	120.5 (5.8)	191.9 (22.4)	125.5 (6.2)
Rectum Volume (cm^3^), mean (sd)	NA^e^	56.9 (29.9)	33.9 (13.9)	76.3 (27.7)

a sd, standard deviation.

b TURP, transurethral resection of the prostate.

c PSA, prostate specific antigen.

d BED, biologically effective dose.

e NA, not available.

**Table 3:** Prevalence of radiotherapy toxicity endpoints.

	Change in toxicity grade from baseline^a^			
0	1	2	3
Rectal bleeding				
RAPPER	446 (85%)	64 (12%)	16 (3%)	1 (< 0%)
RADIOGEN	516 (88%)	52 (9%)	16 (3%)	5 (1%)
Gene-PARE	208 (72%)	25 (9%)	49 (17%)	8 (3%)
CCI	110 (73%)	29 (19%)	6 (4%)	6 (4%)

Urinary frequency				
RAPPER	482 (91%)	42 (8%)	3 (1%)	0
RADIOGEN	423 (89%)	46 (10%)	7 (2%)	1(< 1%)
Gene-PARE	173 (68%)	52 (20%)	25 (10%)	5 (2%)
CCI	120 (80%)	24 (16%)	7 (5%)	0

Decreased urine stream				
RAPPER	483 (94%)	18 (4%)	9 (2%)	2 (< 1%)
RADIOGEN	472 (99%)	2 (< 1%)	3 (1%)	1 (< 1%)
Gene-PARE	189 (74%)	34 (13%)	24 (9%)	8 (3%)
CCI	NA	NA	NA	NA

a For the Gene-PARE, CCI and RADIOGEN studies, the post-treatment rectal bleeding grade already accounts for baseline symptoms. For the CCI study, the post-treatment urinary frequency grade already accounts for baseline symptoms.

**Table 4:** Power to detect significant associations among 1564 radiotherapy patients^a^.

		Minor allele frequency					
0.05	0.10	0.15	0.25	0.35	0.50
Per-allele odds ratio	1.15	0	0	0	0	0	0
1.25	0	0	0	1%	1%	1%
1.5	1%	7%	21%	47%	59%	56%
2.0	64%	99%	100%	100%	100%	100%

a Assuming a type I error rate of 5 × 10^− 8^ and a disease prevalence of 20%, which was the approximate average prevalence of the toxicities included in the study.

**Table 5:** Rectal bleeding multivariable analysis results (p < 5 × 10^− 6^).

rsID	Chr	Minor allele	MAF^a^	RAPPER, N = 527		RADIOGEN, N = 589		Gene-PARE, N = 235		CCI, N = 136		Meta-analysis, N = 1487		
OR^b^ (95% CI^c^)	p-Value	OR (95% CI)	p-Value	OR (95% CI)	p-Value	OR (95% CI)	p-Value	OR (95% CI)	p-Value	phet^d^
rs12497518	3p12.3	A	0.47	0.66 (0.45,0.95)	0.025	0.60 (0.41,0.87)	5.44 × 10^− 3^	0.52 (0.33,0.84)	5.67 × 10^− 3^	0.58 (0.34,0.97)	0.035	**0.60 (0.48,0.74)**	**1.41 × 10**^**− 6**^	0.903
rs141044160^e^	23q23	C	0.05	2.62 (1.53,4.48)	7.86 × 10^− 4^	NA^f^	NA	2.77 (1.48,5.21)	1.06 × 10^− 3^	NA	NA	**2.68 (1.78,4.04)**	**2.26 × 10**^**− 6**^	0.892
rs7432328^g^	3p26.1	A	0.06	3.15 (1.78,5.58)	1.34 × 10^− 4^	NA	NA	NA	NA	4.32 (1.40,13.29)	0.011	**3.36 (2.02,5.59)**	**3.23 × 10**^**− 6**^	0.620
rs6999859	8q21.13	G	0.28	1.73 (1.15,2.61)	9.00 × 10^− 3^	2.10 (1.40,3.16)	4.12 × 10^− 4^	1.36 (0.84,2.20)	0.219	1.6 (0.86,2.99)	0.138	**1.73 (1.37,2.17)**	**3.42 × 10**^**− 6**^	0.592
rs4804134	19p13.2	G	0.31	0.63 (0.44,0.91)	0.011	0.48 (0.32,0.71)	1.58 × 10^− 4^	0.69 (0.42,1.12)	0.131	0.68 (0.38,1.22)	0.186	**0.60 (0.48,0.74)**	**3.47 × 10**^**− 6**^	0.621
rs360071	1q42.12	T	0.49	1.69 (1.20,2.40)	2.80 × 10^− 3^	1.77 (1.23,2.54)	2.08 × 10^− 3^	1.43 (0.92,2.23)	0.110	1.40 (0.82,2.38)	0.217	**1.61 (1.32,1.97)**	**3.62 × 10**^**− 6**^	0.835

a MAF, minor allele frequency.

b OR, odds ratio.

c CI, confidence interval.

d phet = p-value from chi-squared test of heterogeneity.

e For rs141044160, RADIOGEN MAF = 0.048, imputation info = 0.749, OR (95% CI) = 0.81, (0.36, 1.81), p-value = 0.593 and CCI = Not imputed.

f NA, not available.

g For rs7432328, RADIOGEN MAF = 0.030, imputation info = 0.946, OR (95%CI) = 0.37 (0.08, 1.65), p-value = 0.134; Gene-PARE MAF = 0.041, imputation info = 0.953, OR (95% CI) = 0.91 (0.33, 2.52), p-value = 0.735.

**Table 6:** Urinary frequency multivariable analysis results (p < 5 × 10^− 6^).

rsID	Chr	Minor allele	MAF^a^	RAPPER, N = 527		RADIOGEN, N = 477		Gene-PARE, N = 255		CCI, N = 150		Meta-analysis, N = 1409		
OR^b^ (95% CI^c^)	p-Value	OR (95% CI)	p-Value	OR (95% CI)	p-Value	OR (95% CI)	p-Value	OR (95% CI)	p-Value	phet^d^
rs17599026	5q31.2	T	0.08	2.57 (1.28,5.16)	0.012	3.19 (1.57,6.46)	2.36 × 10^− 3^	8.16 (3.04,21.9)	5.88 × 10^− 6^	1.63 (0.59,4.54)	0.358	**3.12 (2.08,4.69)**	**4.16 × 10**^**− 8**^	0.139
rs11574532	12q13.13	T	0.07	1.70 (0.70,4.33)	0.282	3.69 (1.78,7.68)	8.29 × 10^− 4^	3.57 (1.53,8.35)	3.03 × 10^− 3^	5.76 (1.66,19.99)	5.50 × 10^− 3^	**3.25 (2.08,5.07)**	**2.11 × 10**^**− 7**^	0.420
rs342442	4q22.1	T	0.38	0.39 (0.22,0.68)	3.50 × 10^− 4^	0.56 (0.36,0.89)	0.011	0.51 (0.32,0.8)	2.88 × 10^− 3^	0.62 (0.31,1.25)	0.174	**0.51 (0.39,0.66)**	**3.86 × 10**^**− 7**^	0.700
rs4534636	12p13.31	A	0.20	1.27 (0.71,2.27)	0.429	2.48 (1.54,3.99)	2.35 × 10^− 4^	2.06 (1.25,3.41)	4.31 × 10^− 3^	2.03 (0.97,4.25)	0.0461	**1.96 (1.49,2.59)**	**1.67 × 10**^**− 6**^	0.373
rs8098701	18q21.1	T	0.07	2.93 (1.39,6.15)	7.63 × 10^− 3^	2.75 (1.52,4.97)	1.15 × 10^− 3^	1.70 (0.86,3.34)	0.133	2.53 (0.77,8.28)	0.136	**2.41 (1.68,3.47)**	**2.11 × 10**^**− 6**^	0.684
rs7366282^e^	1q41	C	0.06	2.22 (1.02,4.82)	0.056	4.11 (1.82,9.3)	1.27 × 10^− 3^	3.86 (1.55,9.63)	3.03 × 10^− 3^	NA^f^	NA	**3.20 (1.98,5.16)**	**2.03 × 10**^**− 6**^	0.501
rs10209697	2q36.1	A	0.07	4.09 (1.65,10.1)	4.35 × 10^− 3^	4.32 (2.19,8.5)	5.14 × 10^− 5^	1.53 (0.74,3.14)	0.255	1.36 (0.44,4.27)	0.599	**2.66 (1.77,3.99)**	**2.27 × 10**^**− 6**^	0.093
rs4997823	6q14.1	G	0.35	0.40 (0.22,0.74)	1.90 × 10^− 3^	0.37 (0.2,0.67)	5.69 × 10^− 4^	0.68 (0.42,1.11)	0.118	0.44 (0.20,0.99)	0.040	**0.49 (0.36,0.66)**	**2.35 × 10**^**− 6**^	0.372
rs7356945	6p24.1	T	0.33	2.56 (1.58,4.13)	1.10 × 10^− 4^	1.63 (1.08,2.48)	0.022	1.21 (0.8,1.85)	0.368	2.42 (1.25,4.69)	7.32 × 10^− 3^	**1.74 (1.38,2.21)**	**3.71 × 10**^**− 6**^	0.097
rs6003982	22q11.23	G	0.44	0.67 (0.37,1.19)	0.166	0.46 (0.27,0.79)	3.70 × 10^− 3^	0.39 (0.23,0.67)	4.33 × 10^− 6^	0.60 (0.30,1.19)	0.138	**0.51 (0.38,0.68)**	**4.28 × 10**^**− 6**^	0.549
rs10101158	8q24.3	A	0.44	1.63 (0.95,2.81)	0.074	1.56 (0.98,2.48)	0.063	1.99 (1.3,3.06)	1.24 × 10^− 3^	2.17 (1.14,4.11)	0.015	**1.80 (1.40,2.31)**	**4.39 × 10**^**− 6**^	0.792

a MAF, minor allele frequency.

b OR, odds ratio.

c CI, confidence interval.

d phet = p-value from chi-squared test of heterogeneity.

e rs7366282 was not imputed in CCI.

f NA, not available.

**Table 7:** Decreased stream multivariable analysis results (p < 5 × 10^− 6^).

rsID	Chr	Minor allele	MAF^a^	RAPPER, N = 512		RADIOGEN, N = 478		Gene-PARE, N = 255		Meta-analysis, N = 1245		
OR^b^ (95% CI^c^)	p-Value	OR (95% CI)	p-Value	OR (95% CI)	p-Value	OR (95% CI)	p-Value	phet^d^
rs7720298	5p15.2	G	0.24	2.13 (1.21,3.74)	0.010	3.63 (1.08,12.16)	0.044	3.09 (1.9,5.04)	2.34 × 10^− 6^	**2.71 (1.90,3.86)**	**3.21 × 10**^**− 8**^	0.545
rs17362923^e^	8p23.2	G	0.18	3.60 (1.98,6.55)	3.12 × 10^− 5^	NA^f^	NA	2.18 (1.29,3.66)	3.20 × 10^− 3^	**2.70 (1.83,4.00)**	**6.79 × 10**^**− 7**^	0.459
rs76273496	1q31.3	C	0.07	4.02 (1.83,8.81)	1.31 × 10^− 3^	3.69 (0.66,20.46)	0.169	3.32 (1.43,7.68)	5.13 × 10^− 3^	**3.68 (2.13,6.33)**	**2.71 × 10**^**− 6**^	0.948
rs2203205	23q21.1	C	0.29	1.70 (1.15,2.52)	0.010	2.35 (0.97,5.74)	0.054	1.71 (1.26,2.31)	5.87 × 10^− 4^	**1.74 (1.38,2.20)**	**2.86 × 10**^**− 6**^	0.790
rs144596911	3q28	A	0.09	4.41 (2.09,9.28)	2.38 × 10^− 4^	1.01 (0.09,11.12)	0.995	3.28 (1.44,7.45)	4.78 × 10^− 3^	**3.60 (2.11,6.17)**	**2.94 × 10**^**− 6**^	0.493
rs62091368	18p11.32	A	0.07	6.78 (2.26,20.37)	1.26 × 10^− 3^	6.96 (1.71,28.3)	0.015	2.66 (1.08,6.57)	0.037	**4.36 (2.33,8.14)**	**3.95 × 10**^**− 6**^	0.334
rs141342719	5q23.3	T	0.12	5.39 (2.43,11.94)	5.09 × 10^− 5^	1.35 (0.23,7.9)	0.748	2.77 (1.27,6.07)	0.012	**3.50 (2.05,5.95)**	**3.97 × 10**^**− 6**^	0.273
rs673783	18p11.32	G	0.31	2.88 (1.52,5.44)	1.06 × 10^− 3^	2.28 (0.68,7.6)	0.183	2.28 (1.33,3.91)	3.24 × 10^− 3^	**2.49 (1.69,3.67)**	**4.33 × 10**^**− 6**^	0.853

a MAF, minor allele frequency.

b OR, odds ratio.

c CI, confidence interval.

d phet = p-value from chi-squared test of heterogeneity.

e For rs17362923, RADIOGEN MAF = 0.182; imputation info = 0.980. This SNP had a very large standard error in the RADIOGEN dataset, and so it was excluded from meta-analysis.

f NA, not available.

**Table 8:** Overall toxicity (STAT) multivariable analysis results (p < 5 × 10^− 6^).

rsID	Chr	Minor allele	MAF^a^	RAPPER, N = 527		RADIOGEN, N = 474		Gene-PARE, N = 209		CCI, N = 136		Meta-analysis, N = 1,346		
Beta (95% CI^b^)	p-value	Beta (95% CI)	p-value	Beta (95% CI)	p-value	Beta (95% CI)	p-value	Beta (95% CI)	p-value	phet^c^
rs11230328	11q12.2	G	0.27	0.23 (0.06,0.40)	7.96 × 10^− 3^	0.45 (0.29,0.61)	3.50 × 10^− 8^	0.20 (− 0.03,0.43)	0.083	0.22 (− 0.09,0.53)	0.170	0.31 (0.21,0.41)	6.27 × 10^− 10^	0.154
rs147596965^d^	10p14	T	0.07	NA^e^	NA	0.75 (0.47,1.03)	2.73 × 10^− 7^	NA	NA	0.44 (− 0.04,0.92)	0.072	0.67 (0.43,0.91)	6.19 × 10^− 8^	0.150
rs77530448	2q31.1	G	0.09	0.07 (− 0.18,0.33)	0.576	0.51 (0.31,0.71)	4.01 × 10^− 7^	0.41 (0.12,0.70)	5.60 × 10^− 3^	0.29 (− 0.13,0.70)	0.179	0.36 (0.23,0.49)	7.36 × 10^− 8^	0.060
rs4906759^f^	15q12	T	0.06	0.34 (− 0.02,0.71)	0.063	0.86 (0.54,1.18)	2.28 × 10^− 7^	0.32 (− 0.08,0.72)	0.110	NA	NA	0.55 (0.34,0.75)	1.55 × 10^− 7^	0.047
rs71610881^g^	4p15.2	A	0.06	0.70 (0.42,0.97)	1.30 × 10^− 6^	NA	NA	NA	NA	0.36 (− 0.06,0.79)	0.098	0.60 (0.36,0.83)	5.41 × 10^− 7^	0.198
rs141799618^h^	19q13.2	G	0.06	0.33 (0.02,0.65)	0.039	0.50 (0.26,0.74)	5.17 × 10^− 5^	NA	NA	0.45 (− 0.04,0.94)	0.072	0.44 (0.26,0.62)	1.22 × 10^− 6^	0.698
rs2842169	10q26.2	C	0.10	0.33 (0.12,0.53)	1.73 × 10^− 3^	0.24 (0.07,0.41)	7.54 × 10^− 3^	0.28 (− 0.02,0.58)	0.067	0.39 (− 0.02,0.81)	0.065	0.28 (0.17,0.40)	1.45 × 10^− 6^	0.863
rs11219068	11q24.1	A	0.15	0.38 (0.17,0.59)	3.56 × 10^− 4^	0.18 (− 0.01,0.37)	0.062	0.16 (− 0.11,0.43)	0.252	0.49 (0.18,0.79)	2.25 × 10^− 3^	0.28 (0.17,0.40)	1.74 × 10^− 6^	0.204
rs8075565	17p13.2	T	0.13	0.37 (0.16,0.59)	6.43 × 10^− 4^	0.33 (0.14,0.52)	5.91 × 10^− 4^	0.23 (− 0.03,0.49)	0.080	− 0.01 (− 0.33,0.30)	0.933	0.28 (0.16,0.39)	2.20 × 10^− 6^	0.217
rs6535028	4q28.3	T	0.10	0.22 (0.02,0.43)	0.031	0.33 (0.14,0.52)	8.62 × 10^− 4^	0.42 (0.11,0.73)	8.13 × 10^− 3^	0.16 (− 0.19,0.51)	0.374	0.29 (0.17,0.40)	2.70 × 10^− 6^	0.615
rs4775602	15q21.1	C	0.16	0.12 (− 0.04,0.28)	0.135	0.27 (0.12,0.42)	5.54 × 10^− 4^	0.40 (0.17,0.63)	7.39 × 10^− 4^	0.11 (− 0.23,0.44)	0.525	0.23 (0.13,0.32)	3.02 × 10^− 6^	0.240
rs60481745	1p13.3	A	0.10	0.20 (− 0.07,0.46)	0.149	0.39 (0.14,0.64)	2.69 × 10^− 3^	0.65 (0.22,1.08)	3.11 × 10^− 3^	0.57 (0.08,1.05)	0.022	0.37 (0.22,0.53)	3.53 × 10^− 6^	0.260
rs7829759	8p23.2	A	0.11	0.46 (0.23,0.69)	1.29 × 10^− 4^	0.22 (− 0.02,0.46)	0.078	0.33 (0.02,0.64)	0.042	0.26 (− 0.20,0.72)	0.264	0.33 (0.19,0.47)	3.84 × 10^− 6^	0.551
rs79604958^i^	15q24.2	C	0.07	NA	NA	0.35 (0.04,0.66)	0.024	0.44 (0.10,0.78)	0.013	0.77 (0.34,1.21)	6.41 × 10^− 4^	0.47 (0.27,0.68)	4.33 × 10^− 6^	0.289
rs10770857	12p13.31	A	0.37	0.18 (0.05,0.31)	6.02 × 10^− 3^	0.14 (0.02,0.26)	0.017	0.15 (− 0.03,0.33)	0.097	0.28 (0.05,0.51)	0.018	0.17 (0.10,0.25)	4.98 × 10^− 6^	0.774
rs12591436	15q25.1	G	0.34	0.23 (0.09,0.37)	1.56 × 10^− 3^	0.16 (0.04,0.28)	8.79 × 10^− 3^	0.14 (− 0.07,0.35)	0.178	0.20 (− 0.05,0.46)	0.124	0.18 (0.10,0.26)	5.66 × 10^− 6^	0.885

a MAF, minor allele frequency.

b CI, confidence interval.

c phet = p-value from chi-squared test of heterogeneity.

d For rs147596965, RAPPER MAF = 0.045, info = 0.619, beta (95%CI) = − 0.14 (− 0.53, 0.25), p-value = 0.478; Gene-PARE MAF = 0.044, info = 0.662, beta (95% CI) = − 0.31 (− 0.83, 0.20), p-value = 0.237.

e NA, not available.

f For rs4906759, CCI MAF = 0.040, info = 0.604, beta (95% CI) = − 0.71 (− 1.37, − 0.05), p-value = 0.035.

g For rs71610881, RADIOGEN MAF = 0.040, info = 0.986, beta (95% CI) = 0.04 (− 0.24, 0.33), p-value = 0.761; Gene-PARE MAF = 0.022, info = 0.995, beta (95% CI) = − 0.07 (− 0.77, 0.63), p-value = 0.841.

h For rs141799618, Gene-PARE MAF = 0.037, info = 0.780, beta (95% CI) = − 0.27, (− 0.89, 0.35), p-value = 0.395.

i For rs79604958, RAPPER MAF = 0.045, info = 0.499, beta (95% CI) = 0.60 (0.20,1.00), p = 0.0036.

**Table 9:** Ordinal logistic regression results showing association between top SNPs and toxicity grade.

Toxicity endpoint	SNP	RAPPER		RADIOGEN		Gene-PARE		CCI		Meta-analysis		
OR^a^ (95% CI^b^)	p-Value	OR (95% CI)	p-Value	OR (95% CI)	p-Value	OR (95% CI)	p-Value	OR (95% CI)	p-Value	phet
Rectal bleeding	rs12497518	0.66 (0.45,0.95)	0.023	0.60 (0.41,0.87)	5.41 × 10^− 3^	0.52 (0.33,0.83)	4.37 × 10^− 3^	0.58 (0.34,0.96)	0.033	0.60 (0.48,0.73)	9.80 × 10^− 7^	0.894
rs141044160	2.54 (1.52,4.26)	8.70 × 10^− 4^	NA^c^	NA	2.62 (1.52,4.52)	7.79 × 10^− 4^	NA	NA	2.58 (1.77,3.75)	7.47 × 10^− 7^	0.942
rs6999859	1.74 (1.16,2.62)	8.37 × 10^− 3^	2.10 (1.40,3,14)	3.87 × 10^− 4^	1.38 (0.86,2.22)	0.190	1.51 (0.82,2.75)	0.183	1.70 (1.37,2.13)	2.40 × 10^− 6^	0.567
rs4804134	0.64 (0.44,0.91)	0.012	0.47 (0.32,0.70)	1.20 × 10^− 4^	0.68 (0.42,1.10)	0.113	0.69 (0.39,1.22)	0.209	0.60 (0.48,0.74)	3.15 × 10^− 6^	0.572
rs360071	1.69 (1.19,2.38)	2.83 × 10^− 3^	1.74 (1.21,2.49)	2.72 × 10^− 3^	1.43 (0.92,2.22)	0.108	1.40 (0.83,2.38)	0.213	1.60 (1.31,1.95)	4.34 × 10^− 6^	0.851
rs7432328	2.88 (1.67,4.98)	5.29 × 10^− 4^	NA	NA	NA	NA	3.97 (1.38,11.39)	0.012	3.09 (1.90,5.01)	5.29 × 10^− 6^	0.598
Urinary frequency	rs17599026	2.56 (1.28,5.12)	0.012	3.77 (1.79,7.64)	6.97 × 10^− 4^	4.07 (1.96,8.64)	1.71 × 10^− 4^	1.84 (0.64,4.93)	0.250	3.09 (2.11,4.53)	8.06 × 10^− 9^	0.542
rs11574532	1.65 (0.65,4.14)	0.308	3.40 (1.64,6.86)	0.002	2.62 (1.23,5.53)	0.013	4.44 (1.41,13.87)	0.012	2.79 (1.84,4.24)	1.41 × 10^− 6^	0.534
rs342442	0.39 (0.22,0.68)	3.57 × 10^− 4^	0.56 (0.35,0.88)	9.88 × 10^− 3^	0.54 (0.35,0.83)	4.48 × 10^− 3^	0.63 (0.31,1.23)	0.183	0.52 (0.40,0.67)	5.57 × 10^− 7^	0.685
rs7366282	2.44 (1.11,5.36)	0.036	3.69 (1.62,8.08)	2.40 × 10^− 3^	3.80 (1.69,8.40)	1.44 × 10^− 3^	NA	NA	3.25 (2.05,5.14)	4.60 × 10^− 7^	0.681
rs4534636	1.28 (0.71,2.28)	0.417	2.50 (1.55,4.03)	2.05 × 10^− 4^	2.07 (1.29,3.34)	2.57 × 10^− 3^	2.18 (1.06,4.49)	0.035	2.00 (1.53,2.62)	4.75 × 10^− 7^	0.361
rs8098701	3.01 (1.44,6.31)	6.05 × 10^− 3^	2.59 (1.44,4.54)	1.79 × 103	1.46 (0.75,2.74)	0.252	2.90 (0.87,8.97)	0.080	2.28 (1.60,3.24)	4.40 × 10^− 6^	0.426
rs10101158	1.62 (0.94,2.78)	0.077	1.55 (0.98,2.46)	0.064	1.96 (1.31,2.94)	9.06 × 10^− 4^	2.06 (1.12,3.93)	0.020	1.78 (1.39,2.27)	3.67 × 10^− 6^	0.830
rs10209697	3.88 (1.58,9.53)	5.55 × 10^− 3^	3.86 (1.98,7.40)	1.25 × 10^− 4^	1.75 (0.86,3.45)	0.119	1.38 (0.41,4.04)	0.584	2.63 (1.78,3.90)	1.38 × 10^− 6^	0.196
rs6003982	0.65 (0.37,1.17)	0.145	0.47 (0.27,0.80)	4.98 × 103	0.39 (0.23,0.65)	2.48 × 10^− 4^	0.61 (0.30,1.18)	0.144	0.50 (0.38,0.67)	2.74 × 10^− 6^	0.557
rs4997823	0.40 (0.22,0.74)	1.89 × 10^− 3^	0.35 (0.19,0.64)	4.02 × 10^− 4^	0.72 (0.44,1.15)	0.170	0.46 (0.20,0.98)	0.044	0.50 (0.37,0.67)	3.74 × 10^− 6^	0.279
rs7356945	2.52 (1.57,4.04)	1.21 × 10^− 4^	1.60 (1.06,2.43)	0.026	1.26 (0.84,1.89)	0.258	2.37 (1.25,4.63)	8.03 × 10^− 3^	1.74 (1.38,2.19)	2.77 × 10^− 6^	0.122
Decreased stream	rs7720298	2.20 (1.25,3.88)	7.45 × 10^− 3^	3.38 (1.00,11.37)	0.058^d^	3.10 (1.96,4.90)	9.29 × 10^− 7^	NA	NA	2.75 (1.96,3.88)	6.52 × 10^− 9^	0.618
rs17362923	3.58 (1.97,6.51)	3.28 × 10^− 5^	NA	NA	1.96 (1.20,3.20)	7.75 × 10^− 3^	NA	NA	2.50 (1.71,3.65)	2.15 × 10^− 6^	0.126
rs76273496	3.96 (1.83,8.59)	1.31 × 10^− 3^	3.25 (0.65,16.14)	0.191^d^	2.94 (1.37,6.32)	7.72 × 10^− 3^	NA	NA	3.39 (2.02,5.68)	3.45 × 10^− 6^	0.865
rs2203205	1.71 (1.16,2.54)	8.72 × 10^− 3^	2.28 (0.94,5.53)	0.063^d^	1.63 (1.22,2.19)	1.13 × 10^− 3^	NA	NA	1.69 (1.35,2.13)	5.35 × 10^− 6^	0.785
rs141342719	5.11 (2.33,11.21)	7.29 × 10^− 5^	1.32 (0.22,7.88)	0.762^d^	2.58 (1.22,5.45)	0.015	NA	NA	3.28 (1.96,5.52)	6.96 × 10^− 6^	0.270
rs673783	2.83 (1.50,5.34)	1.26 × 10^− 3^	2.14 (0.66,6.93)	0.210^d^	2.32 (1.38,3.89)	1.52 × 10^− 3^	NA	NA	2.47 (1.69,3.61)	3.36 × 10^− 6^	0.865
rs62091368	5.93 (2.14,16.41)	1.62 × 10^− 3^	6.85 (1.70,27.60)	0.016^d^	2.81 (1.17,6.73)	0.024	NA	NA	4.29 (2.36,7.80)	1.89 × 10^− 6^	0.422
rs144596911	4.41 (2.10,9.24)	2.24 × 10^− 4^	1.07 (0.10,12.00)	0.954^d^	3.44 (1.56,7.59)	2.60 × 10^− 3^	NA	NA	3.69 (2.18,6.25)	1.20 × 10^− 6^	0.534

a OR, odds ratio.

b CI, confidence interval.

c NA, not available.

d Adjusted for age and biologically effective dose (BED).

## Appendix A Supplementary Data

Supplementary material. Image 1

## References

[bb0005] Alemozaffar M., Regan M.M., Cooperberg M.R., Wei J.T., Michalski J.M., Sandler H.M., Hembroff L., Sadetsky N., Saigal C.S., Litwin M.S., Klein E., Kibel A.S., Hamstra D.A., Pisters L.L., Kuban D.A., Kaplan I.D., Wood D.P., Ciezki J., Dunn R.L., Carroll P.R., SANDA M.G. (2011). Prediction of erectile function following treatment for prostate cancer. JAMA.

[bb9000] Barnett G.C., West C.M., Dunning A.M., Elliott R.M., Coles C.E., Pharoah P.D., Burnet N.G. (2009). Normal tissue reactions to radiotherapy: towards tailoring treatment dose by genotype. Nat Rev Cancer.

[bb0010] Barnett G.C., Coles C.E., Elliott R.M., Baynes C., Luccarini C., Conroy D., Wilkinson J.S., Tyrer J., Misra V., Platte R., Gulliford S.L., Sydes M.R., Hall E., Bentzen S.M., Dearnaley D.P., Burnet N.G., Pharoah P.D., Dunning A.M., West C.M. (2012). Independent validation of genes and polymorphisms reported to be associated with radiation toxicity: a prospective analysis study. Lancet Oncol..

[bb0015] Barnett G.C., West C.M., Coles C.E., Pharoah P.D., Talbot C.J., Elliott R.M., Tanteles G.A., Symonds R.P., Wilkinson J.S., Dunning A.M., Burnet N.G., Bentzen S.M. (2012). Standardized total average toxicity score: a scale- and grade-independent measure of late radiotherapy toxicity to facilitate pooling of data from different studies. Int. J. Radiat. Oncol. Biol. Phys..

[bb0020] Barnett G.C., Thompson D., Fachal L., Kerns S., Talbot C., Elliott R.M., Dorling L., Coles C.E., Dearnaley D.P., Rosenstein B.S., vega A., Symonds P., Yarnold J., Baynes C., Michailidou K., Dennis J., Tyrer J.P., Wilkinson J.S., Gomez-Caamano A., Tanteles G.A., Platte R., Mayes R., Conroy D., Maranian M., Luccarini C., Gulliford S.L., Sydes M.R., Hall E., Haviland J., Misra V., Titley J., Bentzen S.M., Pharoah P.D., Burnet N.G., Dunning A.M., West C.M. (2014). A genome wide association study (GWAS) providing evidence of an association between common genetic variants and late radiotherapy toxicity. Radiother. Oncol..

[bb9005] Barnett G.C., Kerns S.L., Noble D.J., Dunning A.M., West C.M., Burnet N.G. (2015). Incorporating Genetic Biomarkers into Predictive Models of Normal Tissue Toxicity. Clin Oncol (R Coll Radiol).

[bb0025] Barrett J.C., Fry B., Maller J., Daly M.J. (2005). Haploview: analysis and visualization of LD and haplotype maps. Bioinformatics.

[bb0030] Bentzen S.M., Constine L.S., Deasy J.O., Eisbruch A., Jackson A., Marks L.B., Ten Haken R.K., Yorke E.D. (2010). Quantitative analyses of normal tissue effects in the clinic (QUANTEC): an introduction to the scientific issues. Int. J. Radiat. Oncol. Biol. Phys..

[bb0035] Burnet N.G., Elliott R.M., Dunning A., West C.M. (2006). Radiosensitivity, radiogenomics and RAPPER. Clin. Oncol. (R. Coll. Radiol.).

[bb0040] Chatterjee N., Shi J., Garcia-Closas M. (2016). Developing and evaluating polygenic risk prediction models for stratified disease prevention. Nat. Rev. Genet..

[bb0045] Christensen R. (2013). Ordinal-regression models for ordinal data. R package version.

[bb0050] Cohn L.D., Becker B.J. (2003). How meta-analysis increases statistical power. Psychol. Methods.

[bb0055] Corradin O., Scacheri P.C. (2014). Enhancer variants: evaluating functions in common disease. Genitourin. Med..

[bb0070] Davison B.J., Gleave M.E., Goldenberg S.L., Degner L.F., Hoffart D., Berkowitz J. (2002). Assessing information and decision preferences of men with prostate cancer and their partners. Cancer Nurs..

[bb0075] Dearnaley D., Syndikus I., Sumo G., Bidmead M., Bloomfield D., Clark C., Gao A., Hassan S., Horwich A., Huddart R., Khoo V., Kirkbride P., Mayles H., Mayles P., Naismith O., Parker C., Patterson H., Russell M., Scrase C., South C., Staffurth J., Hall E. (2012). Conventional versus hypofractionated high-dose intensity-modulated radiotherapy for prostate cancer: preliminary safety results from the CHHiP randomised controlled trial. Lancet Oncol..

[bb0080] Desmet F.O., Hamroun D., Lalande M., Collod-Beroud G., Claustres M., Beroud C. (2009). Human splicing finder: an online bioinformatics tool to predict splicing signals. Nucleic Acids Res..

[bb0085] Edvardsen H., Landmark-Hoyvik H., Reinertsen K.V., Zhao X., Grenaker-Alnaes G.I., Nebdal D., Syvanen A.C., Rodningen O., Alsner J., Overgaard J., Borresen-Dale A.L., Fossa S.D., Kristensen V.N. (2013). SNP in TXNRD2 associated with radiation-induced fibrosis: a study of genetic variation in reactive oxygen species metabolism and signaling. Int. J. Radiat. Oncol. Biol. Phys..

[bb0090] Encode Project Consortium (2012). An integrated encyclopedia of DNA elements in the human genome. Nature.

[bb0095] Escudier E., Duquesnoy P., Papon J.F., Amselem S. (2009). Ciliary defects and genetics of primary ciliary dyskinesia. Paediatr. Respir. Rev..

[bb0100] Evangelou E., Ioannidis J.P. (2013). Meta-analysis methods for genome-wide association studies and beyond. Nat. Rev. Genet..

[bb0105] Fachal L., Gomez-Caamano A., Sanchez-Garcia M., Carballo A., Peleteiro P., Lobato-Busto R., Carracedo A., Vega A. (2012). TGFbeta1 SNPs and radio-induced toxicity in prostate cancer patients. Radiother. Oncol..

[bb0110] Fachal L., Gomez-Caamano A., Barnett G.C., Peleteiro P., Carballo A.M., Calvo-Crespo P., Kerns S.L., Sanchez-Garcia M., Lobato-Busto R., Dorling L., Elliott R.M., Dearnaley D.P., Sydes M.R., Hall E., Burnet N.G., Carracedo A., Rosenstein B.S., West C.M., Dunning A.M., Vega A. (2014). A three-stage genome-wide association study identifies a susceptibility locus for late radiotherapy toxicity at 2q24.1. Nat. Genet..

[bb0120] Genomes Project, C., Abecasis G.R., Auton A., Brooks L.D., Depristo M.A., Durbin R.M., Handsaker R.E., Kang H.M., Marth G.T., Mcvean G.A. (2012). An integrated map of genetic variation from 1,092 human genomes. Nature.

[bb0125] GTEX Consortium (2013). The genotype-tissue expression (GTEx) project. Nat. Genet..

[bb0130] Guerra J.L., Gomez D., Wei Q., Liu Z., Wang L.E., Yuan X., Zhuang Y., Komaki R., Liao Z. (2012). Association between single nucleotide polymorphisms of the transforming growth factor beta1 gene and the risk of severe radiation esophagitis in patients with lung cancer. Radiother. Oncol..

[bb0135] Heemsbergen W.D., Peeters S.T., Koper P.C., Hoogeman M.S., Lebesque J.V. (2006). Acute and late gastrointestinal toxicity after radiotherapy in prostate cancer patients: consequential late damage. Int. J. Radiat. Oncol. Biol. Phys..

[bb0140] Hnisz D., Abraham B.J., Lee T.I., Lau A., Saint-Andre V., Sigova A.A., Hoke H.A., Young R.A. (2013). Super-enhancers in the control of cell identity and disease. Cell.

[bb0145] Ho A.Y., Atencio D.P., Peters S., Stock R.G., Formenti S.C., Cesaretti J.A., Green S., Haffty B., Drumea K., Leitzin L., Kuten A., Azria D., Ozsahin M., Overgaard J., Andreassen C.N., Trop C.S., Park J., Rosenstein B.S. (2006). Genetic predictors of adverse radiotherapy effects: the Gene-PARE project. Int. J. Radiat. Oncol. Biol. Phys..

[bb0150] Howie B.N., Donnelly P., Marchini J. (2009). A flexible and accurate genotype imputation method for the next generation of genome-wide association studies. PLoS Genet..

[bb0155] Howlader N., A. N., Krapcho M., Garshell J., Neyman N., SF A., CL K., M Y., Ruhl J., Tatalovich Z., Cho H., Mariotto A., DR L., HS C., EJ F., KA C. (2013). SEER Cancer Statistics Review, 1975–2010, based on November 2012 SEER Data Submission.

[bb0160] International Hapmap, Consortium (2003). The international HapMap project. Nature.

[bb0165] Kerns S.L., Ostrer H., Stock R., Li W., Moore J., Pearlman A., Campbell C., Shao Y., Stone N., Kusnetz L., Rosenstein B.S. (2010). Genome-wide association study to identify single nucleotide polymorphisms (SNPs) associated with the development of erectile dysfunction in African-American men after radiotherapy for prostate cancer. Int. J. Radiat. Oncol. Biol. Phys..

[bb0170] Kerns S.L., Stock R., Stone N., Buckstein M., Shao Y., Campbell C., Rath L., de Ruysscher D., Lammering G., Hixson R., Cesaretti J., Terk M., Ostrer H., Rosenstein B.S. (2013). A 2-stage genome-wide association study to identify single nucleotide polymorphisms associated with development of erectile dysfunction following radiation therapy for prostate cancer. Int. J. Radiat. Oncol. Biol. Phys..

[bb0175] Kerns S.L., Stock R.G., Stone N.N., Blacksburg S.R., Rath L., Vega A., Fachal L., Gomez-Caamano A., de Ruysscher D., Lammering G., Parliament M., Blackshaw M., Sia M., Cesaretti J., Terk M., Hixson R., Rosenstein B.S., Ostrer H. (2013). Genome-wide association study identifies a region on chromosome 11q14.3 associated with late rectal bleeding following radiation therapy for prostate cancer. Radiother. Oncol..

[bb0180] Kerns S.L., Stone N.N., Stock R.G., Rath L., Ostrer H., Rosenstein B.S. (2013). A 2-stage genome-wide association study to identify single nucleotide polymorphisms associated with development of urinary symptoms after radiotherapy for prostate cancer. J. Urol..

[bb0185] Kerns S.L., de Ruysscher D., Andreassen C.N., Azria D., Barnett G.C., Chang-Claude J., Davidson S., Deasy J.O., Dunning A.M., Ostrer H., Rosenstein B.S., West C.M., Bentzen S.M. (2014). STROGAR - STrengthening the Reporting Of Genetic Association studies in Radiogenomics. Radiother. Oncol..

[bb0190] Kneebone A., Mameghan H., Bolin T., Berry M., Turner S., Kearsley J., Graham P., Fisher R., Delaney G. (2004). Effect of oral sucralfate on late rectal injury associated with radiotherapy for prostate cancer: a double-blind, randomized trial. Int. J. Radiat. Oncol. Biol. Phys..

[bb0195] Little J., Higgins J.P., Ioannidis J.P., Moher D., Gagnon F., von Elm E., Khoury M.J., Cohen B., Davey-Smith G., Grimshaw J., Scheet P., Gwinn M., Williamson R.E., Zou G.Y., Hutchings K., Johnson C.Y., Tait V., Wiens M., Golding J., van Duijn C., Mclaughlin J., Paterson A., Wells G., Fortier I., Freedman M., Zecevic M., King R., Infante-Rivard C., Stewart A., Birkett N. (2009). Strengthening the reporting of genetic association studies (STREGA): an extension of the STROBE statement. Hum. Genet..

[bb0200] Lopez Guerra J.L., Wei Q., Yuan X., Gomez D., Liu Z., Zhuang Y., Yin M., Li M., Wang L.E., Cox J.D., Liao Z. (2011). Functional promoter rs2868371 variant of HSPB1 associates with radiation-induced esophageal toxicity in patients with non-small-cell lung cancer treated with radio(chemo)therapy. Radiother. Oncol..

[bb0205] Marchini J., Howie B., Myers S., Mcvean G., Donnelly P. (2007). A new multipoint method for genome-wide association studies by imputation of genotypes. Nat. Genet..

[bb0210] Mccarthy M.I., Abecasis G.R., Cardon L.R., Goldstein D.B., Little J., Ioannidis J.P., Hirschhorn J.N. (2008). Genome-wide association studies for complex traits: consensus, uncertainty and challenges. Nat. Rev. Genet..

[bb0215] Michalski J.M., Gay H., Jackson A., Tucker S.L., Deasy J.O. (2010). Radiation dose-volume effects in radiation-induced rectal injury. Int. J. Radiat. Oncol. Biol. Phys..

[bb0220] Pang Q., Wei Q., Xu T., Yuan X., Lopez Guerra J.L., Levy L.B., Liu Z., Gomez D.R., Zhuang Y., Wang L.E., Mohan R., Komaki R., Liao Z. (2013). Functional promoter variant rs2868371 of HSPB1 is associated with risk of radiation pneumonitis after chemoradiation for non-small cell lung cancer. Int. J. Radiat. Oncol. Biol. Phys..

[bb0225] Purcell S., Cherny S.S., Sham P.C. (2003). Genetic power calculator: design of linkage and association genetic mapping studies of complex traits. Bioinformatics.

[bb0230] R Core Development Team (2014). R: a language and environment for statistical computing. R Foundation for Statistical Computing (Vienna, Austria).

[bb0235] Resnick M.J., Koyama T., Fan K.H., Albertsen P.C., Goodman M., Hamilton A.S., Hoffman R.M., Potosky A.L., Stanford J.L., Stroup A.M., van Horn R.L., Penson D.F. (2013). Long-term functional outcomes after treatment for localized prostate cancer. N. Engl. J. Med..

[bb0245] Safwat A., Bentzen S.M., Turesson I., Hendry J.H. (2002). Deterministic rather than stochastic factors explain most of the variation in the expression of skin telangiectasia after radiotherapy. Int. J. Radiat. Oncol. Biol. Phys..

[bb0250] Sanda M.G., Dunn R.L., Michalski J., Sandler H.M., Northouse L., Hembroff L., Lin X., Greenfield T.K., Litwin M.S., Saigal C.S., Mahadevan A., Klein E., Kibel A., Pisters L.L., Kuban D., Kaplan I., Wood D., Ciezki J., Shah N., Wei J.T. (2008). Quality of life and satisfaction with outcome among prostate-cancer survivors. N. Engl. J. Med..

[bb0260] Skol A.D., Scott L.J., Abecasis G.R., Boehnke M. (2006). Joint analysis is more efficient than replication-based analysis for two-stage genome-wide association studies. Nat. Genet..

[bb0265] Stock R.G., Stone N.N., Wesson M.F., Dewyngaert J.K. (1995). A modified technique allowing interactive ultrasound-guided three-dimensional transperineal prostate implantation. Int. J. Radiat. Oncol. Biol. Phys..

[bb0270] Stock R.G., Stone N.N., Cesaretti J.A., Rosenstein B.S. (2006). Biologically effective dose values for prostate brachytherapy: effects on PSA failure and posttreatment biopsy results. Int. J. Radiat. Oncol. Biol. Phys..

[bb0275] Stone N.N., Hong S., Lo Y.C., Howard V., Stock R.G. (2003). Comparison of intraoperative dosimetric implant representation with postimplant dosimetry in patients receiving prostate brachytherapy. Brachytherapy.

[bb0280] Sydes M.R., Stephens R.J., Moore A.R., Aird E.G., Bidmead A.M., Fallowfield L.J., Graham J., Griffiths S., Mayles W.P., Mcguire A., Stanley S., Warrington A.P., Dearnaley D.P., Collaborators R.T. (2004). Implementing the UK Medical Research Council (MRC) RT01 trial (ISRCTN 47772397): methods and practicalities of a randomised controlled trial of conformal radiotherapy in men with localised prostate cancer. Radiother. Oncol..

[bb0285] Syndikus I., Morgan R.C., Sydes M.R., Graham J.D., Dearnaley D.P., Collaborators, M. R. (2010). Late gastrointestinal toxicity after dose-escalated conformal radiotherapy for early prostate cancer: results from the UK Medical Research Council RT01 trial (ISRCTN47772397). Int. J. Radiat. Oncol. Biol. Phys..

[bb0290] Talbot C.J., Tanteles G.A., Barnett G.C., Burnet N.G., Chang-Claude J., Coles C.E., Davidson S., Dunning A.M., Mills J., Murray R.J., Popanda O., Seibold P., West C.M., Yarnold J.R., Symonds R.P. (2012). A replicated association between polymorphisms near TNFalpha and risk for adverse reactions to radiotherapy. Br. J. Cancer.

[bb0295] Taylor A.M., Harnden D.G., Arlett C.F., Harcourt S.A., Lehmann A.R., Stevens S., Bridges B.A. (1975). Ataxia telangiectasia: a human mutation with abnormal radiation sensitivity. Nature.

[bb0300] Uhlen M., Fagerberg L., Hallstrom B.M., Lindskog C., Oksvold P., Mardinoglu A., Sivertsson A., Kampf C., Sjostedt E., Asplund A., Olsson I., Edlund K., Lundberg E., Navani S., Szigyarto C.A., Odeberg J., Djureinovic D., Takanen J.O., Hober S., Alm T., Edqvist P.H., Berling H., Tegel H., Mulder J., Rockberg J., Nilsson P., Schwenk J.M., Hamsten M., von Feilitzen K., Forsberg M., Persson L., Johansson F., Zwahlen M., von Heijne G., Nielsen J., Ponten F. (2015). Proteomics. Tissue-based map of the human proteome. Science.

[bb0305] Viswanathan A.N., Yorke E.D., Marks L.B., Eifel P.J., Shipley W.U. (2010). Radiation dose-volume effects of the urinary bladder. Int. J. Radiat. Oncol. Biol. Phys..

[bb0310] von Elm E., Altman D.G., Egger M., Pocock S.J., Gotzsche P.C., Vandenbroucke J.P., Initiative, S. (2007). The strengthening the reporting of observational studies in epidemiology (STROBE) statement: guidelines for reporting observational studies. Ann. Intern. Med..

[bb0315] West C.M., Barnett G.C. (2011). Genetics and genomics of radiotherapy toxicity: towards prediction. Genome Med..

[bb0320] West C., Rosenstein B.S. (2010). Establishment of a radiogenomics consortium. Radiother. Oncol..

