# Supplementary material for: Meta-analysis of Genome Wide Association Studies Identifies Genetic Markers of Late Toxicity Following Radiotherapy for Prostate Cancer
Source: eBioMedicine. 2016 Jul 20;10:150–63. doi: 10.1016/j.ebiom.2016.07.022 (PMC5036513; doi:10.1016/j.ebiom.2016.07.022)
Supplement: Supplementary file 1 — Supplementary material. [file mmc1.docx]

Figure S1. QQ plots of meta-analysis results. A, results from analysis of urinary frequency; B, results from analysis of decreased urine stream; C results from analysis of rectal bleeding; D, results from analysis of overall toxicity measured by STAT score. Panel B includes only 3 studies because this endpoint was not available in the CCI cohort.


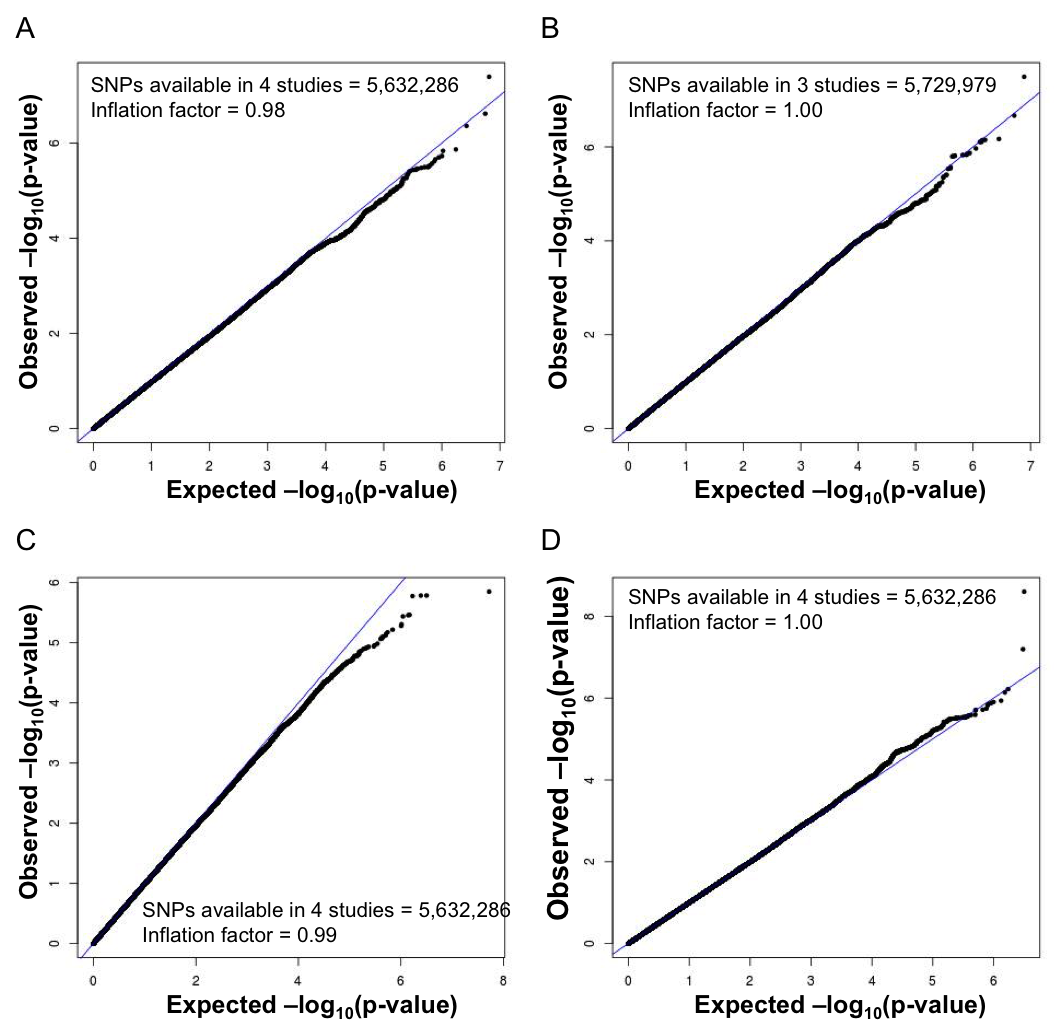


Table S1**.** Toxicity endpoints recorded in the four prostate studies.

| **Toxicity endpoint** | **Study** | **Outcome Scale** | **Grade** |
| --- | --- | --- | --- |
| **Rectal bleeding** | RAPPER | Observer-assigned (RMH^a^) | 0 = No toxicity  1 = Occasional (no treatment)  2 = Moderate (simple OPD^b^ treatment)  3 = Severe (blood transfusion, surgery)  4 = Life-threatening consequences; major urgent intervention indicated |
|  | RADIOGEN, CCI | Observer-assigned (CTCAE^c^ v3.0) | 0 = None  1 = Mild, intervention (other than iron supplements) not indicated  2 = Symptomatic and medical intervention or minor cauterization indicated  3 = Transfusion, interventional radiology, endoscopic, or operative intervention indicated; radiation therapy (i.e., haemostasis of bleeding site)  4 = Life-threatening consequences; major urgent intervention indicated |
|  | Gene-PARE | Observer-assigned (RTOG^d^) | 0 = No toxicity  1 = Slight rectal bleeding (blood streaked stools); no treatment required  2 = Intermittent bleeding  3 = Significant rectal bleeding requiring surgery or cautery or hyperbaric oxygen  4 = Necrosis/perforation/fistula |
| **Proctitis** | RAPPER | Observer-assigned (RTOG) | 0 = No toxicity  1 = Minor symptoms requiring no treatment  2 = Symptoms responding to simple OPD management; lifestyle & PS^e^ unaffected  3 = Distressing symptoms altering lifestyle & PS. Hospitalization for diagnosis or minor surgical intervention may be required  4 = Major surgical intervention (e.g. laparotomy, colostomy) or prolonged hospitalization |
|  | RADIOGEN | Observer-assigned (CTCAE v3.0) | 0 = No toxicity  1 = Rectal discomfort, intervention not indicated  2 = Symptoms not interfering with ADL^f^; medical intervention indicated  3 = Stool incontinence or other symptoms interfering with ADL; operative intervention indicated  4 = Life-threatening consequences (e.g., perforation) |
|  | CCI |  | NA |
|  | Gene-PARE |  | NA |
| **Nocturia** | RAPPER | Patient-reported  (LENT-SOMA^g^) | How many times per night did you most typically get up to urinate?  0 = 0-1 times  1 = 2-3 times  2 = 4-5 times  3 = 6-8 times  4 = >8 times |
|  | RADIOGEN, CCI | Observer-assigned (CTCAE v3.0) | 0 = No toxicity  1 = Increase in frequency or nocturia up to 2 x normal; enuresis  2 = Increase >2 x normal but <hourly  3 = ≥1 x/hr; urgency; catheter indicated |
|  | Gene-PARE | Patient-reported  (IPSS^h^) | In the last month or so, how many times did you most typically get up to urinate from the time you went to bed at night until the time you get up in the morning?  0 = None  1 = 1 time  2 = 2 times  3 = 3 times  4 = 4 times  5 = 5 times or more |
| **Daytime urinary frequency** | RAPPER | Patient-reported  (LENT-SOMA) | Over the last 2 weeks, how frequently do you pass urine?  0 = >4 hour intervals  1 = 3-4 hour intervals  2 = 2-3 hour intervals  3 = 1-2 hour intervals  4 = Hourly |
|  | RADIOGEN, CCI | Observer-assigned (CTCAE v3.0) | 0 = No toxicity  1 = Increase in frequency or nocturia up to 2 x normal; enuresis  2 = Increase >2 x normal but <hourly  3 = ≥1 x/hr; urgency; catheter indicated |
|  | Gene-PARE | Patient-reported  (IPSS) | In the last month or so, how often have you had to urinate again less than 2 hours after you have urinated?  0 = Not at all  1 = Less than 1 time in 5  2 = Less than ½ the time  3 = About ½ the time  4 = More than ½ the time  5 = Almost always |
| **Decreased urine stream** | RAPPER | Patient-reported  (LENT-SOMA) | Is your flow of urine weaker now than before radiotherapy/brachytherapy?  0 = No toxicity  1 = Occasionally weak  2 = Intermittent  3 = Persistent but incomplete  4 = Complete obstruction |
|  | RADIOGEN | Observer-assigned (CTCAE v3.0) | 0 = No toxicity  1 = Hesitancy or dribbling, no significant residual urine; retention occurring during the immediate postoperative period  2 = Hesitancy requiring medication; or operative bladder atony requiring indwelling catheter beyond immediate postoperative period but for <6 weeks  3 = More than daily catheterization indicated; urological intervention indicated (e.g., TURP^i^,  suprapubic tube, urethrotomy)  4 = Life-threatening consequences; organ failure (e.g., bladder rupture); operative intervention requiring organ resection indicated  5 = Death |
|  | CCI |  | NA^j^ |
|  | Gene-PARE | Patient-reported  (IPSS) | In the last month or so, how often have you had a weak urinary stream?  0 = Not at all  1 = Less than 1 time in 5  2 = Less than ½ the time  3 = About ½ the time  4 = More than ½ the time  5 = Almost always |

^a^ RMH = Royal Marsden Hospital; ^b^ OPD = out-patient department; ^c^ CTCAE, Common Terminology Criteria for Adverse Events; ^d^ RTOG, Radiation Therapy Oncology Group; ^e^ PS = performance status; ^f^ ADL = Activities of Daily Living; ^g^ LENT-SOMA, Late effects on Normal Tissues-Subjective Objective Management Analytic; ^h^ IPSS, International Prostate Symptom Score; ^i^ TURP, transurethral resection of the prostate; ^j^ NA, not available.

Table S2. Harmonization of toxicity scoring systems across studies.

| **Toxicity**  **endpoint** | **Study-Specific Toxicity Grade** | | | **Harmonized Grade** |
| --- | --- | --- | --- | --- |
|  | **RAPPER**  **(LENT SOMA^a^)** | **RADIOGEN, CCI**  **(CTCAE^b^ v3.0)** | **Gene-PARE**  **(IPSS^c^)** |  |
| **Nocturia** | 0 | 0 | 0 | 0 |
|  |  |  | 1 |  |
|  | 1 | 1 | 2 | 1 |
|  |  |  | 3 |  |
|  | 2 | 2 | 4 | 2 |
|  | 3 | 3 | 5 | 3 |
|  | 4 |  |  |  |
| **Daytime urinary frequency** | 0 | 0 | 0 | 0 |
|  |  |  | 1 |  |
|  | 1 | 1 | 2 | 1 |
|  | 2 |  | 3 |  |
|  | 3 | 2 | 4 | 2 |
|  | 4 | 3 | 5 | 3 |
| **Decreased urine stream** | 0 | 0 | 0 | 0 |
|  |  |  | 1 |  |
|  | 1 | 1 | 2 | 1 |
|  | 2 | 2 | 3 | 2 |
|  |  |  | 4 |  |
|  | 3 | 3 | 5 | 3 |

^a^ LENT-SOMA, Late effects on Normal Tissues-Subjective Objective Management Analytic; ^b^ CTCAE, Common Terminology Criteria for Adverse Events; ^c^ IPSS, International Prostate Symptom Score

Table S3. Prevalence of each radiotherapy toxicity endpoint in the full patient set from each study.

|  | **Change in toxicity grade from baseline^a^** | | | |
| --- | --- | --- | --- | --- |
|  | **0** | **1** | **2** | **3** |
|  | **Rectal bleeding** | | | |
| RAPPER | 489 (86%) | 65 (11%) | 17 (3%) | 1 (<1%) |
| RADIOGEN^b^ | 608 (89%) | 57 (8%) | 16 (2%) | 6 (1%) |
| Gene-PARE^c^ | 655 (73%) | 83 (9%) | 75 (8%) | 9 (1%) |
| CCI | 113 (73%) | 29 (19%) | 6 (4%) | 6 (4%) |
|  | **Urinary Frequency** | | | |
| RAPPER | 511 (89%) | 50 (9%) | 14 (2%) | 1 (<1%) |
| RADIOGEN | 489 (89%) | 52 (10%) | 7 (1%) | 1 (<1%) |
| Gene-PARE | 546 (68%) | 191 (24%) | 53 (7%) | 18 (2%) |
| CCI | 123 (80%) | 24 (16%) | 7 (5%) | 0 |
|  | **Decreased Urine Stream** | | | |
| RAPPER | 523 (94%) | 22 (4%) | 11 (2%) | 2 (<1%) |
| RADIOGEN | 544 (98%) | 2 (<1%) | 3 (1%) | 1 (<1%) |
| Gene-PARE | 607 (75%) | 122 (15%) | 60 (7%) | 19 (2%) |
| CCI | NA^c^ | NA | NA | NA |

^a^For the Gene-PARE, CCI and RADIOGEN studies, the post-treatment rectal bleeding grade already accounts for baseline symptoms. For the CCI study, the post-treatment urinary frequency grade already account for baseline symptoms. ^b^Two patients lack rectal toxicity data. 140 and 139 patients lack baseline urinary frequency and decreased urine stream toxicity data, respectively. ^c^73 patients lack rectal toxicity data, 52 patients lack baseline urinary frequency and decreased urine stream toxicity data, and 42 patients lack 2-year urinary frequency and decreased urine stream toxicity data. ^d^NA, not available.

Table S4. Simulation modelling of toxicity endpoints: comparison of power to detect association between urinary frequency toxicity and genotype for various minor allele frequencies and odds ratio=1.5.

|  | **Power: % of tests with p-value<5x10^-6^** |
| --- | --- |
| **MAF^a^ = 0.05** |  |
| Ordinal regression | 0.02 |
| Logistic regression 0 vs 1+ | 0.02 |
| Logistic regression 0,1 vs 2+ | 0.002 |
| **MAF = 0.15** |  |
| Ordinal regression | 0.12 |
| Logistic regression 0 vs 1+ | 0.12 |
| Logistic regression 0,1 vs 2+ | 0.004 |
| **MAF = 0.25** |  |
| Ordinal regression | 0.31 |
| Logistic regression 0 vs 1+ | 0.31 |
| Logistic regression 0,1 vs 2+ | 0 |
| **MAF = 0.35** |  |
| Ordinal regression | 0.36 |
| Logistic regression 0 vs 1+ | 0.36 |
| Logistic regression 0,1 vs 2+ | 0.009 |
| **MAF = 0.45** |  |
| Ordinal regression | 0.41 |
| Logistic regression 0 vs 1+ | 0.40 |
| Logistic regression 0,1 vs 2+ | 0.001 |

**^a^**MAF, minor allele frequency

Table S5. Meta-analysis of previously published SNPs on chr2q24.1 and individual toxicity endpoints among the four GWAS: RAPPER, RADIOGEN, Gene-PARE, and CCI.

| **rsID** | **MAF** | **Urinary frequency** | | **Decreased urinary stream** | | **Rectal bleeding** | |
| --- | --- | --- | --- | --- | --- | --- | --- |
|  |  | **OR**  **(95% CI)** | **p-value** | **OR**  **(95% CI)** | **p-value** | **OR**  **(95% CI)** | **p-value** |
| rs10497203 | 0.030 | 1.70 (0.93, 3.11) | 0.085 | 1.76 (0.82, 3.79) | 0.149 | 1.12 (0.61, 2.03) | 0.722 |
| rs7582141 | 0.033 | 1.81 (1.03, 3.21) | 0.041 | 2.16 (1.08, 4.31) | 0.030 | 1.18 (0.67, 2.06) | 0.570 |
| rs6432512 | 0.033 | 1.81 (1.02, 3.20) | 0.042 | 2.15 (1.08, 4.30) | 0.030 | 1.16 (0.66, 2.03) | 0.605 |
| rs264663 | 0.023 | 2.82 (1.17, 6.79) | 0.020 | 1.65 (0.49, 5.56) | 0.417 | 0.88 (0.33, 2.36) | 0.792 |
| rs264651 | 0.036 | 1.86 (1.01, 3.43) | 0.046 | 1.86 (0.86, 4.01) | 0.116 | 1.22 (0.67, 2.23) | 0.518 |
| rs264588 | 0.033 | 1.89 (1.07, 3.32) | 0.027 | 2.16 (1.09, 4.31) | 0.028 | 1.32 (0.77, 2.28) | 0.314 |
| rs264631 | 0.031 | 1.77 (1.00, 3.13) | 0.049 | 2.06 (1.02, 4.14) | 0.042 | 1.23 (0.70, 2.17) | 0.472 |
